# Synthesis and summary of patient‐reported outcome measures to inform the development of a core outcome set in colorectal cancer surgery

**DOI:** 10.1111/codi.13021

**Published:** 2015-10-09

**Authors:** A. G. K. McNair, R. N. Whistance, R. O. Forsythe, J. Rees, J. E. Jones, A. M. Pullyblank, K. N. L. Avery, S. T. Brookes, M. G. Thomas, P. A. Sylvester, A. Russell, A. Oliver, D. Morton, R. Kennedy, D. G. Jayne, R. Huxtable, R. Hackett, S. J. Dutton, M. G. Coleman, M. Card, J. Brown, J. M. Blazeby, the CONSENSUS‐CRC (Core Outcomes and iNformation SEts iN SUrgical Studies – ColoRectal Cancer) Working Group

**Affiliations:** ^1^ Centre for Surgical ResearchSchool of Social and Community MedicineUniversity of BristolBristolUK; ^2^ Severn School of SurgeryUniversity Hospitals Bristol NHS Foundation TrustBristolUK; ^3^ Division of Surgery Head and NeckUniversity Hospitals Bristol NHS Foundation TrustBristolUK; ^4^ Colorectal Cancer Patient RepresentativeNorth Bristol NHS TrustBristolUK; ^5^ Department of General SurgeryNorth Bristol NHS TrustBristolUK; ^6^ Colorectal Surgery UnitUniversity Hospitals Bristol NHS Foundation TrustBristolUK; ^7^ Colorectal Consumer Liaison GroupNational Cancer Research InstituteLondonUK; ^8^ Academic Department of SurgeryUniversity of BirminghamBirminghamUK; ^9^ Department of SurgerySt Mark's Hospital and Academic InstituteHarrowUK; ^10^ Academic Surgical UnitSt James' University Hospital NHS TrustLeedsUK; ^11^ Centre for Ethics in MedicineUniversity of BristolBristolUK; ^12^ Colorectal Network Site Specific GroupAvon, Somerset and Wiltshire Cancer ServicesBristolUK; ^13^ Centre for Statistics in Medicine and Oxford Clinical Trials Research UnitNuffield Department of Orthopaedics, Rheumatology and Musculoskeletal SciencesUniversity of OxfordOxfordUK; ^14^ Department of Colorectal SurgeryPlymouth Hospitals NHS TrustPlymouthUK; ^15^ Clinical Trials Research UnitUniversity of LeedsLeedsUK

**Keywords:** Colorectal cancer, surgery, patient‐reported outcomes, core outcome set, systematic review

## Abstract

**Aim:**

Patient‐reported outcome (PRO) measures (PROMs) are standard measures in the assessment of colorectal cancer (CRC) treatment, but the range and complexity of available PROMs may be hindering the synthesis of evidence. This systematic review aimed to: (i) summarize PROMs in studies of CRC surgery and (ii) categorize PRO content to inform the future development of an agreed minimum ‘core’ outcome set to be measured in all trials.

**Method:**

All PROMs were identified from a systematic review of prospective CRC surgical studies. The type and frequency of PROMs in each study were summarized, and the number of items documented. All items were extracted and independently categorized by content by two researchers into ‘health domains’, and discrepancies were discussed with a patient and expert. Domain popularity and the distribution of items were summarized.

**Results:**

Fifty‐eight different PROMs were identified from the 104 included studies. There were 23 generic, four cancer‐specific, 11 disease‐specific and 16 symptom‐specific questionnaires, and three *ad hoc* measures. The most frequently used PROM was the EORTC QLQ‐C30 (50 studies), and most PROMs (*n *=* *40, 69%) were used in only one study. Detailed examination of the 50 available measures identified 917 items, which were categorized into 51 domains. The domains comprising the most items were ‘anxiety’ (*n *=* *85, 9.2%), ‘fatigue’ (*n *=* *67, 7.3%) and ‘physical function’ (*n *=* *63, 6.9%). No domains were included in all PROMs.

**Conclusion:**

There is major heterogeneity of PRO measurement and a wide variation in content assessed in the PROMs available for CRC. A core outcome set will improve PRO outcome measurement and reporting in CRC trials.

## Introduction

The measurement of patient‐reported outcomes (PROs) has become standard in the assessment of colorectal cancer (CRC) treatments, and their use is recommended by funding and regulatory agencies 1. Many patient‐reported outcome measures (PROMs) have therefore been developed for a variety of purposes 2. Some are generic, and allow comparisons between patients with other conditions (e.g. SF‐36, EQ‐5D), others are designed for patients with cancer (e.g. EORTC QLQ‐C30, FACT‐G), and some are specific for CRC (e.g. EORTC‐CR29, FACT‐C). To add further complexity, each of these PROMs typically consists of a number of questions (items), which are often grouped together to represent similar concepts (scales). For example, two questions regarding activities of daily living and leisure activities in the EORTC QLQ‐C30 measure are grouped into a single ‘role function’ scale. There are therefore a multitude of ways to measure PROs to evaluate treatment for CRC, and this creates problems that may influence the conduct and clinical impact of trials.

Trials may use different PROMs 3, 4 making it impossible to synthesize data across trials or undertake meta‐analyses. The multiplicity of results available from trials means that it is difficult to interpret findings in the context of clinical practice because of a lack of familiarity with the number of measures, scales and items 2. For example, the scale ‘physical function’ exists in several different PROMs, but individual items in these scales vary considerably between questionnaires. This is confusing for clinicians, who may not be aware of the differences between PROMs, and it is likely to limit the meaningful use of the data in practice. Finally, the opportunities for measuring multiple outcomes may lead to selective reporting of significant findings. This can generate bias and influence clinical interpretation of trials 5.

A proposed solution to these issues are ‘core outcome sets’. Core outcomes are the minimum set of outcomes that patients and professionals agree should be measured in all trials of a certain condition 6. They aim to facilitate comparisons between trials and aid meta‐analysis by standardizing outcome measurement, including PROs. The use of core sets may also facilitate the clinical communication of data. Many core outcome sets have now been developed in different clinical areas, including rheumatology 7, paediatrics 8 and obstetrics 9, but not in CRC surgery. This systematic review aims to examine the measurement of PROs in CRC surgical studies, and use the data to inform the development of the core outcome set.

## Method

A systematic review of prospective CRC surgical studies measuring PROs was undertaken to: (i) summarize PRO measurement in CRC surgical studies, and (ii) examine each PROM in detail and categorize analogous concepts into domains to inform the future development of a core outcome set.

### Systematic search and data extraction

This systematic review adhered to a predefined protocol (available on request from the authors). Validated terms relating to ‘surgery’, ‘colorectal cancer’ and ‘prospective studies’ (Table 1) were used to search the OVID SP versions of MEDLINE, EMBASE and the Cochrane Central Register of Controlled Trials. A validated filter for ‘prospective studies’ was used because PRO data are typically collected prospectively. The search was limited to studies conducted in humans aged 18 years and over, reported in the English language between January 2009 and December 2010. Previous reviews have considered PROs of CRC surgery in terms of elderly patients 10, methodological challenges in measuring PROs in CRC 11, laparoscopic surgery 12, long‐term survivors 13, rectal cancer 3 and CRC before 2009 14. The studies identified in these reviews were included. All citations were collated with reference manager 12 (Thomson Reuters, New York city, New York, USA) and the duplicates removed.

**Table 1:** OvidSP version of Medline search strategy

Search criteria	Search terms
Colorectal cancer	1. exp Colonic Neoplasms/
	2. exp Rectal Neoplasms/
	3. ((colorect$ or colon or colonic or rect$) adj3 (cancer$ or tumo?r$ or neoplasm$ or carcinoma$ or adenocarcinoma$ or malignan$)).tw.
	4. or/1‐3
Surgery	1. exp Specialties, Surgical/
	2. surg$.tw.
	3. operat$.tw.
	4. intervention$.tw.
	5. procedur$.tw.
	6. resect$.tw.
	7. or/1‐6
Randomized controlled trials/prospective studies	1. randomized controlled trial.pt.
	2. controlled clinical trial.pt.
	3. randomized controlled trials.sh.
	4. random allocation.sh.
	5. double blind method.sh.
	6. single‐blind method.sh.
	7. or/1‐6
	8. exp animals/not human/
	9. 7 not 8
	10. clinical trial.pt.
	11. exp clinical trials/
	12. (clin$ adj25 trial$).ti,ab.
	13. ((singl$ or doubl$ or trebl$ or tripl$) adj25 (blind$ or mask$)).ti,ab.
	14. placebos.sh.
	15. placebo$.ti,ab.
	16. random$.ti,ab.
	17. research design.sh.
	18. or/10‐17
	19. 18 not 8
	20. 19 not 9
	21. comparative study.sh.
	22. exp evaluation studies/
	23. follow up studies.sh.
	24. prospective studies.sh.
	25. (control$ or prospective$ or volunteer$).ti,ab.
	26. or/21‐25
	27. 26 not 8
	28. 27 not (9 or 20)
	29. 9 or 20 or 28

Titles and abstracts of identified publications were screened by one researcher. If there was uncertainty about the eligibility of a publication the full paper was also accessed. Articles were included if they were original research papers reporting PROs of CRC surgery (curative or palliative), with or without neoadjuvant or adjuvant therapies, or systematic reviews of such publications. PROs were defined as end‐points provided by patients themselves and not interpreted by observers. Studies of nonbiomedical interventions (e.g. alternative medicine), palliative treatments that did not include a surgical component (e.g. palliative chemotherapy), screening studies, treatment of colorectal metastases and molecular and genetic prognostic studies were all excluded. Studies of more than one cancer site or of mixed benign and malignant disease were included provided the data for CRC patients was presented separately from that of the other diseases.

Data extraction included: participant demographics (number, age and gender); treatment received (surgery, neoadjuvant radiotherapy/chemoradiation and adjuvant chemotherapy); treatment intent (curative or palliative); the study design (randomized trial, case–control study, cohort study, cross‐sectional study, prospective case series or other design); the PRO questionnaire used; and the individual items included in each questionnaire. When the individual questionnaires were not available in publications, internet searches and direct contact with authors were used to obtain the information. All data were entered into a Microsoft Access (Microsoft, Redmond, Washington, USA) database to facilitate data management and analyses. The data extraction was checked by a second reviewer (ROF) for a sample of included articles (*n *=* *25) and any disagreements were discussed and resolved with the senior author (JMB).

### Summary of PRO measurement in CRC surgery

The number of publications reporting each PROM was tabulated and descriptive statistics used to summarize PRO measurement. The popularity of PROMs was assessed by comparing their frequency of use in studies. A summary of each PROM is provided in terms of the numbers of items, scales and whether a total score was used. The distribution of items among PROMs was examined by calculating the median number and range of items per PROM. Questionnaires were categorized as: (i) generic (for use in all patients), (ii) cancer specific (for use in all cancer patients), (iii) CRC specific (for use in CRC patients), (iv) symptom specific (to assess a single symptom, e.g. pain), or (v) *ad hoc*.

### Examination of PROs and domain categorization

Individual items from all questionnaires were extracted and formed into a long‐list before categorization into health domains by two researchers (RNW and JR). Both were kept masked as to which PRO questionnaire the items were derived from. Two patient representatives (JEJ and GS) and one consultant colorectal surgeon (AMP) subsequently checked this process. Discrepancies were discussed and resolved with the senior author (JMB).

Categorization was summarized using descriptive statistics to explore the distribution of items and PROMs between domains. The number of items included in each domain was counted, as were the number of PROMs from which they were sourced. The contribution of each source PROM was demonstrated by calculating the median number and range of items included from the measures.

## Results

**Figure 1:**
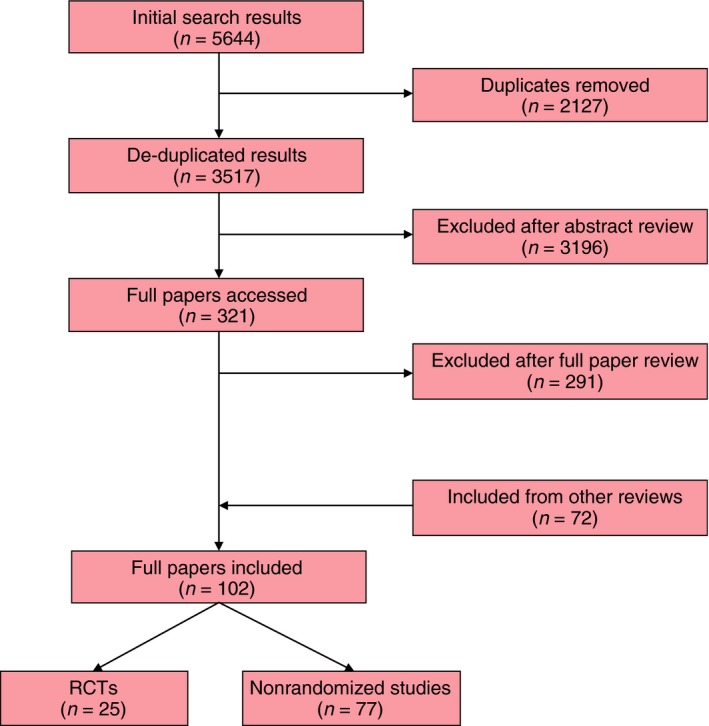
PRISMA (Preferred Reporting Items for Systematic Reviews) diagram of studies considered for the systematic review.

A total of 5644 titles and abstracts were identified, of which 2127 were duplicates. The remaining 3517 were screened and 29 original research articles included. In addition to this, six systematic reviews of PROs in CRC surgery identified a further 72 original research articles (Fig. 1). In total, 102 original publications including 25 randomized controlled trials (25%) and 77 nonrandomized studies (75%) reporting the outcome for 66 386 patients with CRC 15, 16, 17, 18, 19, 20, 21, 22, 23, 24, 25, 26, 27, 28, 29, 30, 31, 32, 33, 34, 35, 36, 37, 38, 39, 40, 41, 42, 43, 44, 45, 46, 47, 48, 49, 50, 51, 52, 53, 54, 55, 56, 57, 58, 59, 60, 61, 62, 63, 64, 65, 66, 67, 68, 69, 70, 71, 72, 73, 74, 75, 76, 77, 78, 79, 80, 81, 82, 83, 84, 85, 86, 87, 88, 89, 90, 91, 92, 93, 94, 95, 96, 97, 98, 99, 100, 101, 102, 103, 104, 105, 106, 107, 108, 109, 110, 111, 112, 113, 114, 115, 116 were included. The studies are summarized in Table 2.

**Table 2:** Summary of included articles

	All studies (*n *=* *102)	Randomized trials (*n *=* *25)	Nonrandomized studies (*n *=* *77)
Number of participants	66 386	7172	59 214
Age range of participants (years)	18–99	29–89	18–99
Number of participating centres (%)			
Single	58 (57)	13 (52)	45 (58)
Multiple	44 (43)	12 (48)	32 (42)
IRB or ethical approval reported (%)	52 (51)	14 (56)	38 (49)
Tumour site (%)			
Colon	10 (9)	2 (8)	7 (9)
Rectum	54 (53)	11 (44)	44 (55)
Mixed colon and rectum	38 (38)	12 (48)	26 (34)
Surgical approach (%)			
Laparoscopy	1 (1)	0	1 (1)
Hand‐assisted laparoscopy	0	0	0
Open	5 (5)	1 (4)	4 (5)
Mixed	13 (12)	9 (36)	5 (6)
Not reported or incomplete information reported	83 (81)	15 (60)	67 (87)
Neoadjuvant treatment^a^ (%)			
Radiotherapy alone	20 (20)	9 (36)	11 (14)
Chemotherapy alone	0	0	0v (0.0)
Chemoradiotherapy	22 (22)	3 (12)	18 (24)
None	7 (7)	1 (4)	6 (8)
Not reported or incomplete information reported	53 (51)	12 (48)	42 (54)
Adjuvant treatment^a^ (%)			
Chemotherapy or chemoradiotherapy	56 (55)	16 (64)	40 (52)
None	0	0	0
Not reported or incomplete information reported	46 (45)	9 (36)	37 (48)
Number of PROMs reported			
1	43	10	33
2	47	12	35
3	6	2	4
4	4	1	3
5	2	0	2

IRB, Institutional Review Board.

a Some studies included patients with or without neoadjuvant therapy, some patients underwent different neoadjuvant or adjuvant treatment within the same study.

### Summary of PROM in CRC surgery

Fifty‐eight different PRO questionnaires were identified and these were reported 184 times in the included publications (Table 3). There were 23 (39.7%) generic questionnaires, four (6.9%) cancer‐specific questionnaires, 11 (19.0%) CRC‐specific questionnaires and 17 (29.3%) symptom‐specific questionnaires. Three *ad hoc* questionnaires (those devised specifically for the study) were not categorized.

**Table 3:** Summary of identified patient‐reported outcome measures (questionnaires) (*n* = 58)

	Number of items	Number of scales	Overall score	Frequency (*n *=* *184)
Name of generic questionnaire (*n* = 23)				
Short Form‐36	36	8	No	21
EuroQol‐5D	6	6	Yes	3
Rotterdam Symptom Checklist	35	4	No	3
Gastrointestinal Quality of Life Index	36	0	Yes	2
Functional Difficulty Index	15	0	Yes	2
Illness Impact Scale	9	0	Yes	2
Visual Analogue Scale (overall health)	1	0	Yes	2
Self‐rated health^a^	–	–	–	1
Freiburger Illness Coping Strategies questionnaire^a^	–	–	–	1
Brief Symptom Inventory‐18	18	3	Yes	1
Constructed Meaning Scale	8	0	Yes	1
Surgical Recovery Score	31	0	Yes	1
Nottingham Health Profile	45	6	Yes	1
Duke Generic Instrument	17	11	Yes	1
Instrumental Activities of Daily Living	7	0	Yes	1
Profile of Moods States	65	6	Yes	1
Health and Activities Limitation Index	8	2	Yes	1
Health Utility Index	7	7	Yes	1
Spitzer Quality of Life Index	5	5	Yes	1
Global Quality of Life^a^	–	–	–	1
Multidimensional Functional Assessment Questionnaire^a^	–	–	–	1
Symptom Experience Scale	24	6	Yes	1
*Ad hoc* satisfaction questionnaire^b^	6	6	Yes	1
Name of cancer‐specific questionnaire (*n* = 4)				
EORTC QLQ‐C30	30	15	No	50
Cancer‐related Health Worries Scale	4	0	Yes	2
Quality of Life – Cancer Survivors	41	4	No	1
Cancer Problems in Living Scale	31	0	Yes	1
Name of disease‐specific questionnaire (*n* = 11)				
EORTC QLQ‐CR38	38	9	No	33
Functional Assessment of Cancer Therapy – Colorectal	37	5	Yes	5
Modified City of Hope Quality of Life – Ostomy	41	6	Yes	2
EORTC QLQ‐CR29	34	4	No	1
University of Padova Bowel Function Questionnaire	8	0	Yes	1
Bowel Function Questionnaire	8	0	Yes	1
Bowel Problems Scale	7	7	No	1
Late Effects Normal Tissue – subjective, objective, management, analytic scale^a^	–	–	–	1
Quality of Life Index for Colostomy Patients	23	3	No	1
Colorectal Cancer Quality of Life	62	4	Yes	1
COloREctal Functional Outcome Questionnaire	26	5	Yes	1
Name of symptom‐specific questionnaire (*n *=* *17)				
International Index of Erectile Function	15	5	Yes	4
Faecal Incontinence Quality of Life Questionnaire	29	4	No	3
Wexner Incontinence Scale	5	0	Yes	3
Visual Analogue Scale (pain)	1	0	Yes	3
Center for Epidemiologic Studies – Depression	20	6	Yes	3
Hospital Anxiety and Depression Scale	14	0	Yes	2
Holschneider Questionnaire	8	0	Yes	1
Internation Index of Erectile Function‐5	5	5	Yes	1
Body Image Questionnaire	10	2	No	1
Body Image Scale	10	0	Yes	1
Faecal Incontinence Scoring System	5	0	Yes	1
Patient Assessment of Constipation Symptom Scale	12	3	Yes	1
Present Pain Intensity Index	1	0	Yes	1
Satisfaction with Sexual Function	1	0	Yes	1
Visual Analogue Scale (wound satisfaction)	1	0	Yes	1
Symptom Distress Scale	15	0	Yes	1
Multidimensional Fatigue Inventory‐20	20	5	No	1
Name of questionnaire (*n *=* *3)				
*Ad hoc* QOL questionnaire A^a^, ^b^	–	–	–	1
*Ad hoc* QOL questionnaire B^a^, ^b^	–	–	–	1
*Ad hoc* QOL questionnaire C^a^, ^b^	–	–	–	1

EORTC, European Organisation for Research and Treatment of Cancer; QOL, quality of life.

a Questionnaire not available.

b PROM not validated.

Most questionnaires were reported only once (*n *=* *40, 69.0%). The most frequently reported were the European Organisation for the Research and Treatment of Cancer (EORTC) QLQ‐C30 (50 studies, 48%), the EORTC QLQ‐CR38 (33 studies, 32%) and the Medical Outcome Study Short Form‐36 (21 studies, 21%). The median number of items per PROM was 14, ranging from one [five PROMs: Visual Analogue Scale (overall, pain and wound satisfaction), Satisfaction with Sexual Function, and Present Pain Intensity Index)] to 65 (the Profile of Mood States). Some 159 scales were evident, and most PROMs (*n *=* *48, 83%) included a total score.

### Examination of PROs and domain categorization

Fifty (86.2%) full questionnaires were available. Reasons for unavailability were inability to obtain the questionnaires from authors or web searches (*n *=* *6) or lack of an English language translation (*n *=* *2). The 50 questionnaires comprised some 917 individual items, and were categorized into 51 domains as described above (Table 4). The full categorization is presented in Table S1. The domains comprising the most items were ‘anxiety’ (*n *=* *85, 9.2%), ‘fatigue’ (*n *=* *67, 7.2%) and ‘physical function’ (*n *=* *63, 6.8%). The disease‐specific domains comprising most items were ‘faecal incontinence’ (*n *=* *53, 5.7%) and ‘stoma problems’ (*n *=* *52, 5.6%). Most domains (*n *=* *27, 53%) contained 10 or more items.

**Table 4:** Summary of domain categorization including number of items per domain, numbers of PROMs and median items per PROM

PRO domain (*n *=* *51)	Number of items (*n *=* *917 (%)	Number of PROMs (*n *=* *50) (%)	Median items per source PROM (range)
Psychological domains			
Anxiety	85 (9.2)	22 (44)	2.5 (1–12)
Fatigue	67 (7.2)	21 (42)	1.0 (1–23)
Depression	47 (5.1)	16 (32)	1.5 (1–12)
Body image	37 (4.0)	13 (26)	1.0 (1–10)
Frustration/irritability	15 (1.6)	7 (14)	1.0 (1–9)
Outlook on life	13 (1.4)	5 (10)	2.0 (1–6)
Self‐esteem	11 (1.2)	6 (12)	2.0 (1–3)
Coping	10 (1.1)	6 (12)	1.0 (1–3)
Spiritual	7 (0.7)	2 (4)	3.5 (3–4)
Regret	5 (0.5)	2 (4)	2.5 (1–4)
Control	3 (0.3)	3 (6)	1.0 (1)
Functional domains			
Physical function	63 (6.8)	19 (38)	1.0 (1–9)
Role function	51 (5.5)	20 (40)	2.0 (1–7)
Social function	50 (5.4)	22 (44)	2.0 (1–8)
Sexual function	44 (4.7)	13 (26)	1.0 (1–15)
Cognitive function	30 (3.2)	14 (28)	1.0 (1–7)
Symptom domains			
Faecal incontinence	53 (5.7)	12 (24)	2.0 (1–27)
Stoma problems	52 (5.6)	5 (10)	7.0 (7–21)
Pain	50 (5.4)	18 (36)	1.5 (1–8)
Insomnia	18 (1.9)	13 (26)	1.0 (1–4)
Appetite/eating problems	17 (1.8)	10 (20)	1.5 (1–3)
Faecal frequency	14 (1.5)	8 (16)	2.0 (1–3)
Nausea/vomiting	12 (1.3)	8 (16)	1.0 (1–3)
Faecal Urgency	11 (1.2)	8 (16)	1.0 (1–2)
Flatulence or gas	11 (1.2)	7 (14)	1.0 (1–3)
Treatment problems	11 (1.2)	7 (14)	1.0 (1–3)
Rectal blood or mucus	10 (1.1)	8 (16)	1.0 (1–2)
Bloating	7 (0.7)	6 (12)	1.0 (1–2)
Diarrhoea	7 (0.7)	7 (14)	1.0 (1)
Tenesmus	7 (0.7)	4 (8)	2.0 (1–2)
Constipation	6 (0.6)	5 (10)	1.0 (1–2)
Shortness of breath	5 (0.5)	5 (10)	1.0 (1)
Urinary frequency	5 (0.5)	3 (6)	2.0 (1–2)
Faint or dizzy	4 (0.4)	4 (8)	1.0 (1)
Hair problems	4 (0.4)	4 (8)	1.0 (1)
Discrimination	3 (0.3)	3 (6)	1.0 (1)
Dry mouth	3 (0.3)	3 (6)	1.0 (1)
Menstruation	3 (0.3)	3 (6)	1.0 (1)
Taste	3 (0.3)	3 (6)	1.0 (1)
Duration of bowel movement	2 (0.2)	2 (4)	1.0 (1)
Dyspepsia	2 (0.2)	2 (4)	1.0 (1)
Dysphagia	2 (0.2)	1 (2)	2.0 (2)
Dysuria	1 (0.1)	1 (2)	1.0 (1)
Urinary incontinence	1 (0.1)	1 (2)	1.0 (1)
Global domains			
Global quality of life	12 (1.3)	9 (18)	1.0 (1–2)
Self‐care	10 (1.1)	10 (20)	1.0 (1)
Financial	8 (0.9)	5 (10)	1.0 (1–4)
Satisfaction with care	6 (0.6)	1 (2)	6.0 (6)
Information needs	1 (0.1)	1 (2)	1.0 (1)

There was little evidence of consistency between PROMs. No domains were measured in all the PROMs. The two domains that were best represented were ‘anxiety’ and ‘social function’, each measured by 22 (44%) PROMs. Otherwise, most domains (*n *=* *39, 76%) were measured by less than a quarter of PROMs, highlighting further heterogeneity. There were two domains with a high median number of items included per PROM: ‘stoma problems’, which contained 52 items from only five PROMs (median seven items per PROM) and ‘satisfaction with care’, which featured six items from just one PROM. This may reflect specialization of PROMs, with some measures focusing on very specific concepts.

## Discussion

This systematic review aimed to summarize PRO measurement in current CRC surgical studies and categorize PRO items into analogous concepts to inform the development of a core outcome set. There was evidence of significant heterogeneity of PRO measurement in the included studies. Fifty‐eight different PROMs were used to assess patient experience of colorectal surgery. Most (*n *=* *40, 69.0%) were only ever used once, and even the most common (EORTC QLQ‐C30) was measured in less than half of the studies. PROMs also varied greatly in terms of their content, with some as simple as a single item while others included up to 65. Most (52%) PROMs were not designed to be specific to CRC surgery or symptoms thereof, and although this may bring benefits in terms of comparison between diseases they may not be sensitive enough to issues that are of specific importance to patients with CRC. Over 900 individual questionnaire items were evident from 50 PROMs, and through a rigorous process, these were categorized into 51 ‘health domains’. This demonstrated a further lack of consistency, with no domains being measured in all the PROMs, and most health domains only being measured by less than a quarter of PROMs. All of this highlights potentially major questions for evidence synthesis and clinical interpretation of results in studies of CRC surgery, and demonstrates the need for a standardized core outcome set.

Other studies have highlighted the problem with outcome heterogeneity for clinical and PRO data. A recently published systematic review identified 194 studies of CRC surgery that measured 766 different clinical outcomes, with no single outcome reported in all 117. Even considering a seemingly simple outcome such as mortality, there were over 84 different ways in which this was defined and measured. The same problem has been highlighted in studies of oesophageal cancer surgery 118, where a review of 122 articles reported 210 unique complications and 10 different measures of operative mortality, and breast reconstruction following mastectomy for cancer 119, which identified 134 studies reporting 950 unique complications. Problems with the multiplicity of PRO measures have also been described previously in oesophageal surgery 120, but there is no evidence of this issue in trials of CRC surgery.

This study is the largest systematic review of PROs in CRC and was conducted with rigorous methodology, but there are some limitations. The review covers published CRC studies in English up until 2010. A more exhaustive search over a more recent period of time, or inclusion of unpublished data or non‐English publications may have yielded further PROs, but all the most commonly used PROs were captured by these inclusion criteria and extending the review would have probably only identified additional rare PROs. The categorization process could be criticized as arbitrary, but efforts were made to objectify the process. First, two researchers categorized the questionnaire items independently, each blinded to the other. Second, categorization was checked for face validity by a patient representative. Finally, there has been full disclosure of the categorization in this article to allow scientific scrutiny of the process.

Having identified all the potential patient reported health domains measured in CRC surgical studies, the next phase of this research is to gain a consensus on which outcomes it is essential to measure in all trials. Recommended methods to achieve this have been defined by the international Core Outcome Measurement in Effectiveness Trials (COMET) group 6. Domains will be combined with clinical outcomes generated from a previous systematic review 117 to create an exhaustive long‐list of all potential outcomes. Key stakeholders, including patients and professionals, will then consider the importance of these outcomes and undertake a prioritization exercise called the Delphi process. This will allow the outcomes of lesser importance to be discarded from the core set. Finally, when the number of outcomes has been reduced, face‐to‐face meeting will be conducted to allow for debate about their relative merits before the final core set is agreed.

In conclusion, this systematic review of CRC surgery demonstrated significant heterogeneity of PRO measurement that may hinder comparisons between studies, limit meta‐analysis and allow outcome reporting bias. A long‐list of patient reported ‘health domains’ was generated using robust methodology to inform the development of a core outcome set.

## Supporting information


**Table S1.** Full categorization of patient reported outcome items.
