# Supplementary material for: Synthesis and summary of patient‐reported outcome measures to inform the development of a core outcome set in colorectal cancer surgery
Source: Colorectal Dis. 2015 Oct 9;17(11):O217–29. doi: 10.1111/codi.13021 (PMC4744711; doi:10.1111/codi.13021)
Supplement: Supplementary file 1 — Table S1. Full categorization of patient reported outcome items. [file CODI-17-O217-s001.docx]

**Table 5 (supporting information, not in published article): Full categorization of patient reported outcome items**

| ID | Item | New domain |
| --- | --- | --- |
| 21 | Did you feel tense? | Anxiety |
| 22 | Did you worry? | Anxiety |
| 46 | Were you worried about your health in the future? | Anxiety |
| 81 | Were you worried about your health in the future? | Anxiety |
| 82 | Have you worried about your Body Image? | Anxiety |
| 121 | I feel nervous | Anxiety |
| 122 | I worry about dying | Anxiety |
| 123 | I worry that my condition will get worse | Anxiety |
| 181 | How often during the past 2 weeks have you been nervous or anxious about your illness? | Anxiety |
| 206 | I feel tense or 'wound up' | Anxiety |
| 208 | I get a sort of frightened feeling as if something awful is about to happen | Anxiety |
| 210 | Worrying thoughts go through my mind | Anxiety |
| 212 | I can sit at ease and feel relaxed | Anxiety |
| 214 | I get a sort of frightened feeling like 'butterflies' in the stomach | Anxiety |
| 216 | I feel restless as I have to be on the move | Anxiety |
| 218 | I get sudden feelings of panic | Anxiety |
| 275 | Have you been a very nervous person? | Anxiety |
| 277 | Have you felt calm and peaceful? | Anxiety |
| 334 | DURING THE PAST 7 DAYS, how much were you distressed by: Nervousness or shakiness inside | Anxiety |
| 338 | DURING THE PAST 7 DAYS, how much were you distressed by: Suddenly scared for no reason | Anxiety |
| 341 | DURING THE PAST 7 DAYS, how much were you distressed by: Feeling fearful | Anxiety |
| 344 | DURING THE PAST 7 DAYS, how much were you distressed by: Numbness or tingling in parts of your body | Anxiety |
| 347 | DURING THE PAST 7 DAYS, how much were you distressed by: Feeling tense or keyed up | Anxiety |
| 348 | DURING THE PAST 7 DAYS, how much were you distressed by: Spells of terror or panic | Anxiety |
| 349 | DURING THE PAST 7 DAYS, how much were you distressed by: Feeling nervous when you are left alone | Anxiety |
| 350 | DURING THE PAST 7 DAYS, how much were you distressed by: Feeling so restless you couldn’t sit still | Anxiety |
| 450 | Do you fear the future? | Anxiety |
| 455 | Are you nervous or stressed? | Anxiety |
| 508 | ANXIETY / DEPRESSION - I am not anxious or depressed; I am slightly anxious or depressed; I am moderately anxious or depressed; I am sverely anxious or depressed; I am extremely anxious or depressed | Anxiety |
| 542 | Do you have a sense of inner peace? | Anxiety |
| 548 | How much anxiety do you have? | Anxiety |
| 549 | Are you fearful that your disease will come back? | Anxiety |
| 557 | I'm feeling on edge. | Anxiety |
| 581 | Worry is keeping me awake at night. | Anxiety |
| 609 | Nervousness | Anxiety |
| 620 | Nervousness, tension, anxiety or depression | Anxiety |
| 622 | Trouble with hands sweating and feeling damp and clammy | Anxiety |
| 623 | Heart beating hard even when not exercising or working hard | Anxiety |
| 644 | I worry about my cancer coming back | Anxiety |
| 645 | I am sometimes concerned that symptoms I experience may indicate the recurrence of cancer | Anxiety |
| 646 | I worry about future diagnostic tests | Anxiety |
| 647 | I worry about another type of cancer | Anxiety |
| 657 | I felt fearful. | Anxiety |
| 669 | Describe HOW YOU FEEL RIGHT NOW: Tense | Anxiety |
| 683 | Describe HOW YOU FEEL RIGHT NOW: On edge | Anxiety |
| 687 | Describe HOW YOU FEEL RIGHT NOW: Panicky | Anxiety |
| 689 | Describe HOW YOU FEEL RIGHT NOW: Relaxed | Anxiety |
| 693 | Describe HOW YOU FEEL RIGHT NOW: Uneasy | Anxiety |
| 694 | Describe HOW YOU FEEL RIGHT NOW: Restless | Anxiety |
| 701 | Describe HOW YOU FEEL RIGHT NOW: Nervous | Anxiety |
| 708 | Describe HOW YOU FEEL RIGHT NOW: Anxious | Anxiety |
| 712 | Describe HOW YOU FEEL RIGHT NOW: Desperate | Anxiety |
| 727 | Describe HOW YOU FEEL RIGHT NOW: Carefree | Anxiety |
| 728 | Describe HOW YOU FEEL RIGHT NOW: Terrified | Anxiety |
| 731 | Describe HOW YOU FEEL RIGHT NOW: Uncertain about things | Anxiety |
| 758 | How distressing were the following aspects of your illness and treatment? Initial diagnosis | Anxiety |
| 759 | How distressing were the following aspects of your illness and treatment? Cancer treatments (i.e. chemotherapy, radiation, or surgery) | Anxiety |
| 760 | How distressing were the following aspects of your illness and treatment? Time since my treatment was completed | Anxiety |
| 761 | How much anxiety do you have? | Anxiety |
| 763 | To what extent are you fearful of: Future diagnostic tests | Anxiety |
| 764 | To what extent are you fearful of: A second cancer | Anxiety |
| 765 | To what extent are you fearful of: Recurrence of your cancer | Anxiety |
| 766 | To what extent are you fearful of: Spreading (metastasis) of your cancer | Anxiety |
| 778 | How much uncertainty do you feel about your future? | Anxiety |
| 784 | EMOTION - 1 Generally happy and free from worry. 2 Occasionally fretful, angry, irritable, anxious, depressed, or suffering “night terrors”. 3 Often fretful, angry, irritable, anxious, depressed, or suffering “night terrors”. 4 Almost always fretful, angry, irritable, anxious, depressed. 5 Extremely fretful, angry, irritable, anxious, or depressed usually requiring hospitalisation or psychiatric institutional care. | Anxiety |
| 795 | During the past 7 days about how much were you distressed or bothered by: Nervousness or shakiness inside | Anxiety |
| 796 | During the past 7 days about how much were you distressed or bothered by: Being suddenly scared for no reason | Anxiety |
| 797 | During the past 7 days about how much were you distressed or bothered by: Feeling fearful | Anxiety |
| 798 | During the past 7 days about how much were you distressed or bothered by: Feeling tense or keyed up | Anxiety |
| 799 | During the past 7 days about how much were you distressed or bothered by: Spells of terror or panic | Anxiety |
| 800 | During the past 7 days about how much were you distressed or bothered by: Feeling so restless you couldn’t sit still | Anxiety |
| 801 | During the past 7 days about how much were you distressed or bothered by: Heavy feelings in arms or legs | Anxiety |
| 809 | During the past 7 days about how much were you distressed or bothered by: Feeling afraid in open spaces or on the streets | Anxiety |
| 831 | Shivering | Anxiety |
| 832 | Tingling of hands and feet | Anxiety |
| 835 | Worrying | Anxiety |
| 837 | Nervousness | Anxiety |
| 838 | Desperate feelings about the future | Anxiety |
| 839 | Tension | Anxiety |
| 840 | Anxiety | Anxiety |
| 849 | Feeling fearful that my illness will return | Anxiety |
| 851 | Concern about relapsing | Anxiety |
| 852 | Fears about the future | Anxiety |
| 858 | Feeling vulnerable | Anxiety |
| 871 | Being concerned about infection and crowds | Anxiety |
| 13 | Have you lacked appetite? | Appetite |
| 134 | I can digest my food well | Appetite |
| 136 | I have a good appetite | Appetite |
| 177 | How often during the past 2 weeks have you found eating to be a pleasure? | Appetite |
| 178 | Because of your illness, to what extent have you restricted the kinds of food you eat? | Appetite |
| 197 | How often during the past 2 weeks have you felt uncomfortable because of your slow speed of eating? | Appetite |
| 439 | Have you eaten certain foods on purpose to make your stools thicker or thinner? | Appetite |
| 440 | Have you purposely avoided certain foods to prevent your stools becoming loose or hard? | Appetite |
| 459 | How is your appetite? | Appetite |
| 465 | To regulate the stool, do you apply some dietary regime? | Appetite |
| 649 | I did not feel like eating; my appetite was poor. | Appetite |
| 742 | To what extent are the following a problem for you: Appetite changes | Appetite |
| 814 | Lack of appetite | Appetite |
| 869 | Eating difficulties | Appetite |
| 909 | Appetite intensity | Appetite |
| 910 | Appetite frequency | Appetite |
| 911 | Appetite distress | Appetite |
| 34 | Did you have a bloated feeling in your abdomen? | Bloating |
| 75 | Did you have a bloated feeling in your abdomen? | Bloating |
| 131 | I have swelling or cramps in my stomach area | Bloating |
| 171 | How often during the past 2 weeks have you had a feeling of fullness in the upper abdomen? | Bloating |
| 172 | How often during the past 2 weeks have you had bloating (sensation of too much gas in the abdomen)? | Bloating |
| 370 | How severe have each of these symptoms been in the last 2 weeks? Bloating in your abdomen | Bloating |
| 460 | Do you mention meteorismus? | Bloating |
| 59 | Have you had blood with your stools? | Blood or Mucus PR |
| 76 | Have you had blood in your stools? | Blood or Mucus PR |
| 77 | Have you had mucus in your stools? | Blood or Mucus PR |
| 203 | How often during the past 2 weeks have you been troubled by blood in the stool? | Blood or Mucus PR |
| 330 | Have you had bleeding with your bowel movements? | Blood or Mucus PR |
| 332 | Have you passed mucus from your rectum? | Blood or Mucus PR |
| 374 | How severe have each of these symptoms been in the last 2 weeks? Rectal bleeding or tearing during or after a bowel movement | Blood or Mucus PR |
| 436 | Have you experienced blood loss during your bowel movements? | Blood or Mucus PR |
| 469 | Did you have black or bloody stool since the surgical intervention? | Blood or Mucus PR |
| 811 | For each of the following, please indicate on average how often in the past month you experienced any amount of accidental bowel leakage: Mucus | Blood or Mucus PR |
| 39 | Have you lost Body Image? | Body Image |
| 43 | Have you felt physically less attractive as a result of your disease or treatment? | Body Image |
| 44 | Have you been feeling less feminine/masculine as a result of your disease or treatment? | Body Image |
| 45 | Have you been dissatisfied with your body? | Body Image |
| 83 | Have you felt physically less attractive as a result of your disease or treatment? | Body Image |
| 84 | Have you been feeling less feminine/masculine as a result of your disease or treatment? | Body Image |
| 85 | Have you been dissatisfied with your body? | Body Image |
| 132 | I am losing Body Image | Body Image |
| 137 | I like the appearance of my body | Body Image |
| 187 | Since becoming ill, have you been troubled by changes in your appearance? | Body Image |
| 215 | I have lost interest in my appearance | Body Image |
| 299 | Are you less satisfied with your body since the operation? | Body Image |
| 300 | Do you think the operation has damaged your body? | Body Image |
| 301 | Do you feel less attractive as a result of your disease or treatment? | Body Image |
| 302 | Do you feel less feminine/masculine as a result of your disease or treatment? | Body Image |
| 303 | Is it difficult to look at yourself naked? | Body Image |
| 304 | On a scale from 1 to 7, how satisfied are you with your (incisional) scar? | Body Image |
| 305 | On a scale from 1 to 7, how would you describe your (incisional) scar? | Body Image |
| 306 | Could you score your own incisional scar on a scale from 1 to 10? | Body Image |
| 309 | Have you been feeling self-conscious about your appearance? | Body Image |
| 310 | Have you felt less physically attractive as a result of your disease or treatment? | Body Image |
| 311 | Have you been dissatisfied with your appearance when dressed? | Body Image |
| 312 | Have you been feeling less feminine/masculine as a result of your disease or treatment? | Body Image |
| 313 | Did you find it difficult to look at yourself naked? | Body Image |
| 314 | Have you been feeling less sexually attractive as a result of your disease or treatment? | Body Image |
| 315 | Did you avoid people because of the way you felt about your appearance? | Body Image |
| 316 | Have you been feeling the treatment has left your body less whole? | Body Image |
| 317 | Have you felt dissatisfied with your body? | Body Image |
| 318 | Have you been dissatisfied with the appearance of your scar? | Body Image |
| 414 | How satisfied are you with the appearance of your scar? Place a vertical mark on the line below to indicate how satisfied you are with your scar. | Body Image |
| 456 | How did your body Body Image change? | Body Image |
| 525 | How satisfied are you with your appearance? | Body Image |
| 756 | Has your illness or treatment caused changes in your appearance? | Body Image |
| 856 | Uncomfortable with changes in my physical appearance | Body Image |
| 921 | Appearance intensity | Body Image |
| 922 | Appearance frequency | Body Image |
| 923 | Appearance distress | Body Image |
| 20 | Have you had difficulty in concentrating on things, like reading a newspaper or watching television? | Cognitive |
| 25 | Have you had difficulty remembering things? | Cognitive |
| 390 | During the last two days I have been forgetful | Cognitive |
| 393 | During the last two days I have been able to concentrate on things | Cognitive |
| 394 | During the last two days my thoughts have wandered easily | Cognitive |
| 399 | During the last two days I have made more mistakes than usual | Cognitive |
| 454 | Is it difficult for you to concentrate when you wish to solve a problem? | Cognitive |
| 528 | How is your ability to remember things? | Cognitive |
| 600 | I have difficulty concentrating | Cognitive |
| 643 | To what extent (if at all) has your capacity to think about or attend to things other than your problems or illness been reduced by your problems or illness? | Cognitive |
| 652 | I had trouble keeping my mind on what I was doing. | Cognitive |
| 673 | Describe how you feel right now: Clear-headed | Cognitive |
| 675 | Describe how you feel right now: Confused | Cognitive |
| 695 | Describe how you feel right now: Unable to concentrate | Cognitive |
| 704 | Describe how you feel right now: Muddled | Cognitive |
| 717 | Describe how you feel right now: Bewildered | Cognitive |
| 718 | Describe how you feel right now: Alert | Cognitive |
| 726 | Describe how you feel right now: Forgetful | Cognitive |
| 754 | How is your present ability to concentrate or to remember things? | Cognitive |
| 785 | COGNITION - 1 Learns and remembers school work normally for age. 2 Learns and remembers school work more slowly than classmates as judged by parents and/or teachers. 3 Learns and remembers very slowly and usually requires special educational assistance. 4 Unable to learn and remember. | Cognitive |
| 833 | Difficulty concentrating | Cognitive |
| 857 | Preoccupation with being ill | Cognitive |
| 860 | Diminished ability to concentrate | Cognitive |
| 902 | Keep my thoughts | Cognitive |
| 903 | Concentrate well | Cognitive |
| 904 | Effort to concentrate | Cognitive |
| 905 | Thoughts easily wander | Cognitive |
| 918 | Concentration intensity | Cognitive |
| 919 | Concentration frequency | Cognitive |
| 920 | Concentration distress | Cognitive |
| 16 | Have you been constipated? | Constipation |
| 60 | Have you had difficulty in moving your bowels? | Constipation |
| 201 | How often during the past 2 weeks have you been troubled by constipation? | Constipation |
| 376 | How severe have each of these symptoms been in the last 2 weeks? Bowel movements that were too hard | Constipation |
| 378 | How severe have each of these symptoms been in the last 2 weeks? Straining or squeezing to try to pass bowel movements | Constipation |
| 745 | To what extent are the following a problem for you: Constipation | Constipation |
| 573 | I feel as if I'm losing control. | Control |
| 752 | Do you feel like you are in control of things in your life? | Control |
| 794 | OUTLOOK – During the past week, the patient: 1) has usually been appearing calm and positive in outlook, accepting and in control of personal circumstances, including surroundings. 2) has sometimes been troubled because not fully in control of personal circumstances or has been having periods of obvious anxiety or depression. 3) has been seriously confused or very frightened or consistently anxious and depressed or unconscious. | Control |
| 119 | I am satisfied with how I am coping with my illness | Coping |
| 120 | I am losing hope in the fight against my illness | Coping |
| 127 | I have accepted my illness | Coping |
| 179 | During the past 2 weeks, how well have you been able to cope with everyday stresses? | Coping |
| 524 | Do you feel like you are in control of things? | Coping |
| 599 | I give up too easily | Coping |
| 749 | How difficult is it for you to cope today as a result of your disease and treatment? | Coping |
| 861 | Feeling helpless | Coping |
| 863 | Feeling dependent | Coping |
| 867 | Feeling isolated | Coping |
| 24 | Did you feel depressed? | Depression |
| 118 | I feel sad | Depression |
| 169 | During the past month, have you felt so sad, discouraged, hopeless, or had so many problems that you wondered if anything was worthwhile? | Depression |
| 180 | How often during the past 2 weeks have you been sad about being ill? | Depression |
| 209 | I can laugh and see the funny side of things | Depression |
| 211 | I feel cheerful | Depression |
| 276 | Have you felt so down in the dumps that nothing could cheer you up? | Depression |
| 279 | Have you felt downhearted and blue? | Depression |
| 281 | Have you been a happy person? | Depression |
| 337 | During the past 7 days, how much were you distressed by: Thoughts of ending your life | Depression |
| 339 | During the past 7 days, how much were you distressed by: Feeling blue | Depression |
| 340 | During the past 7 days, how much were you distressed by: Feeling no interest in things | Depression |
| 345 | During the past 7 days, how much were you distressed by: Feeling hopeless about the future | Depression |
| 351 | During the past 7 days, how much were you distressed by: Feelings of worthlessness | Depression |
| 546 | How much depression do you have? | Depression |
| 553 | Things are getting me down. | Depression |
| 556 | I've forgotten what it's like to enjoy myself. | Depression |
| 566 | The days seem to drag. | Depression |
| 582 | I feel that life is not worth living. | Depression |
| 587 | I wake up feeling depressed. | Depression |
| 608 | Feeling depressed or sad | Depression |
| 650 | I felt that I could not shake off the blues even with help from my family or friends. | Depression |
| 653 | I felt depressed. | Depression |
| 655 | I felt hopeful about the future. | Depression |
| 656 | I thought my life had been a failure. | Depression |
| 659 | I was happy. | Depression |
| 664 | I had crying spells. | Depression |
| 665 | I felt sad. | Depression |
| 672 | Describe how you feel right now: unhappy | Depression |
| 680 | Describe how you feel right now: considerate | Depression |
| 681 | Describe how you feel right now: sad | Depression |
| 685 | Describe how you feel right now: blue | Depression |
| 688 | Describe how you feel right now: hopeless | Depression |
| 692 | Describe how you feel right now: sympathetic | Depression |
| 697 | Describe how you feel right now: helpful | Depression |
| 699 | Describe how you feel right now: discouraged | Depression |
| 703 | Describe how you feel right now: miserable | Depression |
| 710 | Describe how you feel right now: Good-natured | Depression |
| 711 | Describe how you feel right now: gloomy | Depression |
| 715 | Describe how you feel right now: helpless | Depression |
| 751 | How much happiness do you feel? | Depression |
| 762 | How much depression do you have? | Depression |
| 781 | How hopeful do you feel? | Depression |
| 806 | During the past 7 days about how much were you distressed or bothered by: Feeling blue | Depression |
| 808 | During the past 7 days about how much were you distressed or bothered by: Feeling no interest in things | Depression |
| 836 | Depressed mood | Depression |
| 901 | Don't feel like doing anything | Depression |
| 17 | Have you had diarrhea? | Diarrhoea |
| 135 | I have diarrhea (diarrhoea) | Diarrhoea |
| 200 | How often during the past 2 weeks have you been troubled by diarrhoea? | Diarrhoea |
| 221 | consistency of stool | Diarrhoea |
| 327 | In the past week have you had diarrhea or loose watery stools? | Diarrhoea |
| 464 | Stool consistency? | Diarrhoea |
| 539 | Problem with diarrhea? | Diarrhoea |
| 223 | discrimination | Discrimination |
| 422 | Was it difficult to distinguish between passing wind and a bowel movement? | Discrimination |
| 463 | Can you differentiate between stool and gases? | Discrimination |
| 40 | Did you have a dry mouth? | Dry Mouth |
| 78 | Did you have a dry mouth? | Dry Mouth |
| 827 | Dry mouth | Dry Mouth |
| 324 | How long do you spend on a single evacuation? >30, 10-30, <10 minutes | Duration of bowel movement |
| 426 | When you went to the toilet, did your bowel movement require more than 15 min? | Duration of bowel movement |
| 204 | How often during the past 2 weeks have you been troubled by heartburn? | Dyspepsia |
| 823 | Heartburn/belching | Dyspepsia |
| 196 | How often during the past 2 week, have you been troubled by fluid or food coming up into your mouth (Dysphagia)? | Dysphagia |
| 198 | How often during the past 2 weeks have you had trouble swallowing your food? | Dysphagia |
| 33 | Did you have pain when you urinated? | Dysuria |
| 54 | Do you have a stoma (colostomy bag)? | EXCLUDE |
| 86 | Do you have a stoma bag (colostomy/ileostomy)? | EXCLUDE |
| 138 | Do you have an ostomy appliance? | EXCLUDE |
| 250 | The following items are about activities you might do during a typical day. Does your health now limit you in these activities? If so, how much? Yes, Limited a lot; Yes, Limited a little; No, Not limited at all | EXCLUDE |
| 261 | During the past 4 weeks, have you had any of the following problems with your work or other regular daily activities as a result of your physical health? | EXCLUDE |
| 266 | During the past 4 weeks, have you had any of the following problems with your work or other regular daily activities as a result of any emotional problems (such as feeling depressed or anxious)? | EXCLUDE |
| 55 | Did you have frequent bowel movements during the day? | Faecal Frequency |
| 56 | Did you have frequent bowel movements during the night? | Faecal Frequency |
| 97 | (No stoma) Did frequent bowel movements occur during the day? | Faecal Frequency |
| 98 | (No stoma) Did frequent bowel movements occur during the night? | Faecal Frequency |
| 176 | How often during the past 2 weeks have you been troubled by frequent bowel movements? | Faecal Frequency |
| 220 | frequency of stool | Faecal Frequency |
| 365 | Bowel movements > 3/day | Faecal Frequency |
| 367 | Nocturnal evacuation | Faecal Frequency |
| 433 | How many bowel movements have you had during the day? | Faecal Frequency |
| 434 | How many bowel movements have you had during the night? | Faecal Frequency |
| 462 | Number of stool / day? | Faecal Frequency |
| 924 | Bowel pattern intensity | Faecal Frequency |
| 925 | Bowel pattern frequency | Faecal Frequency |
| 926 | Bowel pattern distress | Faecal Frequency |
| 58 | Have you had any unintentional release of stools? | Faecal Incontinence |
| 95 | (No stoma) Have you had leakage of stools from your back passage? | Faecal Incontinence |
| 133 | I have control of my bowels | Faecal Incontinence |
| 142 | Due to accidental bowel leakage: I am afraid to go out | Faecal Incontinence |
| 143 | Due to accidental bowel leakage: I avoid visiting friends | Faecal Incontinence |
| 144 | Due to accidental bowel leakage: I avoid staying overnight away from home | Faecal Incontinence |
| 145 | Due to accidental bowel leakage: It is difficult for me to get out and do things like going to a movie or to church | Faecal Incontinence |
| 146 | Due to accidental bowel leakage: I cut down on how much I eat before I go out | Faecal Incontinence |
| 147 | Due to accidental bowel leakage: Whenever I am away from home, I try to stay near a restroom as much as possible | Faecal Incontinence |
| 148 | Due to accidental bowel leakage: It is important to plan my schedule (daily activities) around my bowel pattern | Faecal Incontinence |
| 149 | Due to accidental bowel leakage: I avoid traveling | Faecal Incontinence |
| 150 | Due to accidental bowel leakage: I worry about not being able to get to the toilet in time | Faecal Incontinence |
| 151 | Due to accidental bowel leakage: I feel I have no control over my bowels | Faecal Incontinence |
| 152 | Due to accidental bowel leakage: I can't hold my bowel movement long enough to get to the bathroom | Faecal Incontinence |
| 153 | Due to accidental bowel leakage: I leak stool without even knowing it | Faecal Incontinence |
| 154 | Due to accidental bowel leakage: I try to prevent bowel accidents by staying very near a bathroom | Faecal Incontinence |
| 155 | Due to accidental bowel leakage: I feel ashamed | Faecal Incontinence |
| 156 | Due to accidental bowel leakage: I can not do many of things I want to do | Faecal Incontinence |
| 157 | Due to accidental bowel leakage: I worry about bowel accidents | Faecal Incontinence |
| 158 | Due to accidental bowel leakage: I feel depressed | Faecal Incontinence |
| 159 | Due to accidental bowel leakage: I worry about others smelling stool on me | Faecal Incontinence |
| 160 | Due to accidental bowel leakage: I feel like I am not a healthy person | Faecal Incontinence |
| 161 | Due to accidental bowel leakage: I enjoy life less | Faecal Incontinence |
| 162 | Due to accidental bowel leakage: I have sex less often than I would like to | Faecal Incontinence |
| 163 | Due to accidental bowel leakage: I feel different from other people | Faecal Incontinence |
| 164 | Due to accidental bowel leakage: The possibility of bowel accidents is always on my mind | Faecal Incontinence |
| 165 | Due to accidental bowel leakage: I am afraid to have sex | Faecal Incontinence |
| 166 | Due to accidental bowel leakage: I avoid traveling by plane or train | Faecal Incontinence |
| 167 | Due to accidental bowel leakage: I avoid going out to eat | Faecal Incontinence |
| 168 | Due to accidental bowel leakage: Whenever I go someplace new, I specifically locate where the bathrooms are | Faecal Incontinence |
| 205 | How often during the past 2 weeks have you been troubled by uncontrolled stools? | Faecal Incontinence |
| 225 | fecal soiling | Faecal Incontinence |
| 226 | wearing of pads | Faecal Incontinence |
| 227 | use of drugs/enema/lifestyle alteration | Faecal Incontinence |
| 289 | How often during the past 4 weeks have you experienced accidental bowel leakage of gas? | Faecal Incontinence |
| 290 | How often during the past 4 weeks have you experienced minor bowel soiling or seepage? | Faecal Incontinence |
| 291 | How often during the past 4 weeks have you experienced significant accidental leakage of liquid stools? | Faecal Incontinence |
| 292 | How often during the past 4 weeks have you experienced significant accidental leakage of solid stools? | Faecal Incontinence |
| 293 | How often during the past 4 weeks have you experienced how often has this accidental leakage affected your QoL? | Faecal Incontinence |
| 294 | How often do you have incontinence to solids? Never = 0 (never), Rarely = <l/month, Sometimes = <l/week,_>l/month, Usually = <l/day, _>l/week, Always = _>l/day | Faecal Incontinence |
| 295 | How often do you have incontinence to fluids? Never = 0 (never), Rarely = <l/month, Sometimes = <l/week,_>l/month, Usually = <l/day, _>l/week, Always = _>l/day | Faecal Incontinence |
| 319 | How often are you incontinent of faeces (/week)? >=2, <2–0, Never | Faecal Incontinence |
| 320 | Do you use pads? Always, Occasionally, Never | Faecal Incontinence |
| 416 | Have you unintentionally passed liquid stools during the day? | Faecal Incontinence |
| 417 | Have you unintentionally passed liquid stools during the night? | Faecal Incontinence |
| 418 | Have you unintentionally passed solid stools during the day? | Faecal Incontinence |
| 419 | Have you unintentionally passed solid stools during the night? | Faecal Incontinence |
| 420 | Have you had a smear of faeces in your underwear during the day? | Faecal Incontinence |
| 421 | Have you had a smear of faeces in your underwear, pyjamas or night-gown at the end of the night? | Faecal Incontinence |
| 423 | Have you used something to protect your underwear, such as sanitary towels, pantyliners or nappies? | Faecal Incontinence |
| 535 | Problem with odor? | Faecal Incontinence |
| 812 | For each of the following, please indicate on average how often in the past month you experienced any amount of accidental bowel leakage: Liquid Stool | Faecal Incontinence |
| 813 | For each of the following, please indicate on average how often in the past month you experienced any amount of accidental bowel leakage: Solid Stool | Faecal Incontinence |
| 57 | Did you feel the urge to move your bowels without actually producing any stools? | Faecal Urgency |
| 199 | How often during the past 2 weeks have you been troubled by urgent bowel movements? | Faecal Urgency |
| 222 | impulse to defecate/sensibility | Faecal Urgency |
| 224 | warning period | Faecal Urgency |
| 321 | How long can you defer defaecation? <10, 10–30, >30 minutes | Faecal Urgency |
| 328 | Have you had a sense of urgency that you move your bowels? | Faecal Urgency |
| 333 | Have you had the feeling that you have an urge to move your bowels, but have nothing to pass? | Faecal Urgency |
| 361 | Urgency | Faecal Urgency |
| 379 | How severe have each of these symptoms been in the last 2 weeks? Feeling like you had to pass a bowel movement but couldn't (false alarm) | Faecal Urgency |
| 424 | If you needed to go urgently, did you have trouble stopping your bowel movement for longer than 15 min? | Faecal Urgency |
| 425 | Have you had a false alarm? (i.e. a need to go without a bowel movement)? | Faecal Urgency |
| 335 | During the past 7 days, how much were you distressed by: Faintness or dizziness | Faint or Dizzy |
| 626 | Fainting spells, dizziness, sick feelings | Faint or Dizzy |
| 677 | Describe how you feel right now: Shaky | Faint or Dizzy |
| 830 | Dizziness | Faint or Dizzy |
| 10 | Did you need to rest? | Fatigue |
| 12 | Have you felt weak? | Fatigue |
| 18 | Were you tired? | Fatigue |
| 104 | I have a lack of energy | Fatigue |
| 184 | How often during the past 2 weeks have you been tired or fatigued? | Fatigue |
| 213 | I feel as if I am slowed down | Fatigue |
| 274 | Did you feel full of pep? | Fatigue |
| 278 | Did you have a lot or energy? | Fatigue |
| 280 | Did you feel worn out? | Fatigue |
| 282 | Did you feel tired? | Fatigue |
| 346 | During the past 7 days, how much were you distressed by: Feeling weak in parts of your body | Fatigue |
| 382 | During the last two days I have been feeling drained | Fatigue |
| 383 | During the last two days I start things without difficulty then get tired | Fatigue |
| 384 | During the last two days I have been feeling energetic | Fatigue |
| 385 | During the last two days I have had trouble paying attention | Fatigue |
| 386 | During the last two days I have been feeling worn out | Fatigue |
| 387 | During the last two days I have been feeling refreshed | Fatigue |
| 388 | During the last two days my body has been feeling heavy all over | Fatigue |
| 389 | During the last two days I have been feeling vigorous | Fatigue |
| 395 | During the last two days I lack the energy to do things I normally do | Fatigue |
| 396 | During the last two days I have been feeling fatigued | Fatigue |
| 397 | During the last two days I have had the energy to do lots of things | Fatigue |
| 398 | During the last two days physically, I have felt tired | Fatigue |
| 401 | During the last two days I have been feeling lively | Fatigue |
| 403 | During the last two days, I have had enough energy to bath/wash | Fatigue |
| 404 | During the last two days, I have had enough energy to dress | Fatigue |
| 405 | During the last two days, I have had enough energy to do household chores | Fatigue |
| 406 | During the last two days, I have had enough energy to cook | Fatigue |
| 407 | During the last two days, I have had enough energy to work | Fatigue |
| 408 | During the last two days, I have had enough energy to visit or socialize with family and friends | Fatigue |
| 409 | During the last two days, I have had enough energy to engage in leisure or recreational activities | Fatigue |
| 410 | During the last two days, I have had enough energy to shop or do errands | Fatigue |
| 411 | During the last two days, I have had enough energy to walk | Fatigue |
| 412 | During the last two days, I have had enough energy to exercise other than walk | Fatigue |
| 452 | Are you weak? | Fatigue |
| 453 | Do you easily get tired? | Fatigue |
| 530 | How much fatigue do you have? | Fatigue |
| 551 | I'm tired all the time. | Fatigue |
| 562 | Everything is an effort. | Fatigue |
| 576 | I soon run out of energy. | Fatigue |
| 607 | Getting tired easily | Fatigue |
| 625 | Weakness, tiring easily, no energy | Fatigue |
| 654 | I felt that everything I did was an effort. | Fatigue |
| 667 | I could not get “going.” | Fatigue |
| 671 | Describe how you feel right now: worn out | Fatigue |
| 674 | Describe how you feel right now: lively | Fatigue |
| 678 | Describe how you feel right now: listless | Fatigue |
| 686 | Describe how you feel right now: energetic | Fatigue |
| 696 | Describe how you feel right now: fatigued | Fatigue |
| 707 | Describe how you feel right now: exhausted | Fatigue |
| 713 | Describe how you feel right now: sluggish | Fatigue |
| 716 | Describe how you feel right now: weary | Fatigue |
| 723 | Describe how you feel right now: Full of pep | Fatigue |
| 732 | Describe how you feel right now: bushed | Fatigue |
| 741 | To what extent are the following a problem for you: Fatigue | Fatigue |
| 792 | HEALTH – During the last week, the patient: 1) has been appearing to feel well or reporting feeling ‘great’ most of the time. 2) has been lacking energy or not feeling entirely ‘up to par’ more than just occasionally. 3) has been feeling very ill or ‘lousy’, seeming weak and washed out most of the time or was unconscious. | Fatigue |
| 805 | During the past 7 days about how much were you distressed or bothered by: Feeling weak in parts of your body | Fatigue |
| 815 | Tiredness | Fatigue |
| 816 | Lack of energy | Fatigue |
| 850 | Fatigue, loss of strength | Fatigue |
| 886 | Fit | Fatigue |
| 887 | Tired | Fatigue |
| 888 | Rested | Fatigue |
| 889 | Tire easily | Fatigue |
| 912 | Fatigue intensity | Fatigue |
| 913 | Fatigue frequency | Fatigue |
| 914 | Fatigue distress | Fatigue |
| 28 | Has your physical condition or medical treatment caused you financial difficulties? | Financial |
| 520 | How much financial burden results form illness/treatment? | Financial |
| 634 | Managing money - I = Able to manage buying needs (i.e., write checks, pay bills) A = Able to manage daily buying needs, but needs help managing checkbook and/or paying bills D = Unable to handle money | Financial |
| 774 | How much financial burden have you incurred as a result of your illness and treatment? | Financial |
| 864 | Difficulty in meeting my medical expenses | Financial |
| 866 | Being less able to provide for the financial needs of my family | Financial |
| 872 | Difficulty in obtaining adequate insurance | Financial |
| 874 | Not being able to change jobs for fear of losing my health insurance coverage | Financial |
| 37 | Were you bothered by gas (flatulence)? | Flatulence or Gas |
| 38 | Did you belch? | Flatulence or Gas |
| 94 | (No stoma) Have you had unintentional release of gas/flatulence from your back passage? | Flatulence or Gas |
| 173 | How often during the past 2 weeks have you been troubled by excessive passage of gas through the anus? | Flatulence or Gas |
| 174 | How often during the past 2 weeks have you been troubled by strong burping or belching? | Flatulence or Gas |
| 175 | How often during the past 2 weeks have you been troubled by gurgling noises from the abdomen? | Flatulence or Gas |
| 296 | How often do you have incontinence to gas? Never = 0 (never), Rarely = <l/month, Sometimes = <l/week,_>l/month, Usually = <l/day, _>l/week, Always = _>l/day | Flatulence or Gas |
| 297 | How often do you require pads? Never = 0 (never), Rarely = <l/month, Sometimes = <l/week,_>l/month, Usually = <l/day, _>l/week, Always = _>l/day | Flatulence or Gas |
| 415 | Have you unintentionally passed wind? | Flatulence or Gas |
| 536 | Problem with gas? | Flatulence or Gas |
| 810 | For each of the following, please indicate on average how often in the past month you experienced any amount of accidental bowel leakage: Gas | Flatulence or Gas |
| 23 | Did you feel irritable? | Frustration |
| 183 | How often during the past 2 weeks have you been frustrated about your illness? | Frustration |
| 570 | I lose my temper easily these days. | Frustration |
| 648 | I was bothered by things that usually don’t bother me. | Frustration |
| 670 | Describe how you feel right now: angry | Frustration |
| 679 | Describe how you feel right now: peeved | Frustration |
| 684 | Describe how you feel right now: grouchy | Frustration |
| 698 | Describe how you feel right now: annoyed | Frustration |
| 700 | Describe how you feel right now: resentful | Frustration |
| 709 | Describe how you feel right now: Ready to fight | Frustration |
| 714 | Describe how you feel right now: rebellious | Frustration |
| 720 | Describe how you feel right now: furious | Frustration |
| 724 | Describe how you feel right now: Bad-tempered | Frustration |
| 834 | Irritability | Frustration |
| 862 | Feeling angry | Frustration |
| 29 | How would you rate your overall health during the past week? | Global Health |
| 108 | I am bothered by side effects of treatment | Global Health |
| 109 | I feel ill | Global Health |
| 141 | In general, would you say your health is (1 poor to 5 excellent) | Global Health |
| 185 | How often during the past 2 weeks have you felt unwell? | Global Health |
| 248 | In general, would you say your health is: Excellent, Very Good, Good, Fair, Poor | Global Health |
| 249 | Compared to one year ago, how would you rate your health in general now? Much better now than one year ago, Somewhat better now than one year ago, About the same as one year ago, Somewhat worse now than one year ago, Much worse than one year ago | Global Health |
| 285 | I seem to get sick a little easier than other people | Global Health |
| 286 | I am as healthy as anybody I know | Global Health |
| 287 | I expect my health to get worse | Global Health |
| 288 | My health is excellent | Global Health |
| 326 | How would you rate your overall defaecatory function? Poor, Fair, Good | Global Health |
| 473 | How do you judge your condition? | Global Health |
| 474 | How do you judge your health status? | Global Health |
| 509 | We would like to know how good or bad your health is TODAY. This scale is numbered from 0 to 100. 100 means the best health you can imagine. 0 means the worst health you can imagine. Mark an X on the scale to indicate how your health is TODAY. Now, please write the number you marked on the scale in the box below. | Global Health |
| 598 | I am basically a healthy person | Global Health |
| 733 | Would you say that your general health is: 1-Excellent to 5-Poor | Global Health |
| 789 | How do you rate your overall health? | Global Health |
| 859 | Continued major problems with my health | Global Health |
| 30 | How would you rate your overall quality of life during the past week? | Global QoL |
| 126 | I am able to enjoy life | Global QoL |
| 130 | I am content with the quality of my life right now | Global QoL |
| 182 | How often during the past 2 weeks have you been happy with life in general? | Global QoL |
| 207 | I still enjoy the things I used to enjoy | Global QoL |
| 217 | I look forward with enjoyment to things | Global QoL |
| 475 | How do you judge your quality of life? | Global QoL |
| 522 | How much satisfaction/enjoyment in life? | Global QoL |
| 663 | I enjoyed life. | Global QoL |
| 705 | Describe HOW YOU FEEL RIGHT NOW: Cheerful | Global QoL |
| 750 | How good is your quality of life? | Global QoL |
| 753 | How satisfying is your life? | Global QoL |
| 41 | Have you had thin or lifeless hair as a result of your disease or treatment? | Hair Problems |
| 79 | Have you lost hair as a result of your treatment? | Hair Problems |
| 480 | If you had a hair loss, was it disturbing you?? | Hair Problems |
| 825 | Loss of hair | Hair Problems |
| 877 | Not being able to get the information I need about cancer | Information needs |
| 11 | Have you had trouble sleeping? | Insomnia |
| 128 | I am sleeping well | Insomnia |
| 186 | Over the past week, have you woken up in the night? | Insomnia |
| 449 | Do you wake up sometimes because of defecation problem or stoma-care? | Insomnia |
| 534 | Do you have sleep disturbances? | Insomnia |
| 563 | I'm waking up in the early hours of the morning. | Insomnia |
| 572 | I lie awake for most of the night. | Insomnia |
| 579 | It takes me a long time to get to sleep. | Insomnia |
| 583 | I sleep badly at night. | Insomnia |
| 605 | Sleeping | Insomnia |
| 621 | Trouble getting to sleep or staying asleep | Insomnia |
| 658 | My sleep was restless. | Insomnia |
| 744 | To what extent are the following a problem for you: Sleep changes | Insomnia |
| 821 | Difficulties sleeping | Insomnia |
| 853 | Sleep difficulties | Insomnia |
| 915 | Sleep intensity | Insomnia |
| 916 | Sleep frequency | Insomnia |
| 917 | Sleep distress | Insomnia |
| 479 | (Women) Do you have any problem with your period? | Menstruation |
| 747 | To what extent are the following a problem for you: Menstrual changes or fertility | Menstruation |
| 788 | FERTILITY - 1 Able to have children with a fertile spouse. 2 Difficulty in having children with a fertile spouse. 3 Unable to have children with a fertile spouse. | Menstruation |
| 14 | Have you felt nauseated? | Nausea and vomiting |
| 15 | Have you vomited? | Nausea and vomiting |
| 105 | I have nausea | Nausea and vomiting |
| 202 | How often during the past 2 weeks have you been troubled by nausea? | Nausea and vomiting |
| 342 | During the past 7 days, how much were you distressed by: Nausea or upset stomach | Nausea and vomiting |
| 457 | Do you suffer of nausea? | Nausea and vomiting |
| 458 | Are you vomiting? | Nausea and vomiting |
| 746 | To what extent are the following a problem for you: Nausea | Nausea and vomiting |
| 829 | Nausea | Nausea and vomiting |
| 906 | Nausea intensity | Nausea and vomiting |
| 907 | Nausea frequency | Nausea and vomiting |
| 908 | Nausea distress | Nausea and vomiting |
| 352 | I feel cancer is something I will never recover from. | Outlook |
| 353 | I feel cancer is serious, but I will be able to return to life as it was before my cancer experience. | Outlook |
| 354 | I feel cancer has changed my life permanently so it will never be as good again. | Outlook |
| 355 | I feel I have made a complete recovery from my cancer experience. | Outlook |
| 356 | I feel that I am the same person as I was before my cancer experience. | Outlook |
| 358 | I feel that my experience with cancer has made me a better person. | Outlook |
| 451 | How often you think about dying? | Outlook |
| 543 | How hopeful do you feel? | Outlook |
| 547 | How much uncertainty do you feel about your future? | Outlook |
| 855 | Having difficulty in making long-term plans | Outlook |
| 898 | Want to do nice things | Outlook |
| 899 | Dread doing things | Outlook |
| 900 | Lots of plans | Outlook |
| 9 | Have you had pain? | Pain |
| 19 | Did pain interfere with your daily activities? | Pain |
| 35 | Did you have abdominal pain? | Pain |
| 36 | Did you have pain in your buttocks? | Pain |
| 61 | Have your bowel movements been painful? | Pain |
| 72 | Did you have pain when you urinated? | Pain |
| 73 | Did you have abdominal pain? | Pain |
| 74 | Did you have pain in your buttocks/anal area/rectum? | Pain |
| 96 | (No stoma) Have you had sore skin around your anal area? | Pain |
| 107 | I have pain | Pain |
| 170 | How often during the past 2 weeks have you had pain in the abdomen? | Pain |
| 271 | How much bodily pain have you had during the past 4 weeks? | Pain |
| 272 | During the past 4 weeks, how much did pain interfere with your normal work (including both work outside the home and housework)? | Pain |
| 329 | Have you had any tenderness or pain when you move your bowels? | Pain |
| 331 | Have you had abdominal cramping or pain? | Pain |
| 336 | During the past 7 days, how much were you distressed by: Pains in the heart or chest | Pain |
| 368 | How severe have each of these symptoms been in the last 2 weeks? Discomfort in your abdomen | Pain |
| 369 | How severe have each of these symptoms been in the last 2 weeks? Pain in your abdomen | Pain |
| 371 | How severe have each of these symptoms been in the last 2 weeks? Stomach cramps | Pain |
| 372 | How severe have each of these symptoms been in the last 2 weeks? Painful bowel movements | Pain |
| 373 | How severe have each of these symptoms been in the last 2 weeks? Rectal burning during or after a bowel movement | Pain |
| 380 | How bad is your pain? 1=no pain, 2=mild, 3=discomforting, 4=distressing, 5=horrible, 6=excruciating | Pain |
| 413 | How severe is your pain today? Place a vertical mark on the line below to indicate how bad you feel your pain is today. | Pain |
| 435 | Have you had pain during your bowel movements? | Pain |
| 437 | Have you had irritated skin around your anus? | Pain |
| 461 | Do you have abdominal pain? | Pain |
| 468 | Do you sense anal or perineal pain? | Pain |
| 507 | PAIN / DISCOMFORT - I have no pain or discomfort; I have slight pain or discomfort; I have moderate pain or discomfort; I have severe pain or discomfort; I have extreme pain or discomfort | Pain |
| 533 | Do you have aches or pains? | Pain |
| 552 | I have pain at night. | Pain |
| 554 | I have unbearable pain. | Pain |
| 558 | I find it painful to change position. | Pain |
| 569 | I'm in pain when I walk. | Pain |
| 574 | I'm in pain when I'm standing. | Pain |
| 578 | I'm in constant pain | Pain |
| 586 | I'm in pain when going up or down stairs. | Pain |
| 588 | I'm in pain when I'm sitting. | Pain |
| 606 | Hurting or aching in any part of your body | Pain |
| 624 | Pains, aches or swelling in parts of the body | Pain |
| 743 | To what extent are the following a problem for you: Aches or pain | Pain |
| 787 | PAIN - 1 Free of pain and discomfort. 2 Occasional pain. Discomfort relieved by non-prescription drugs or self-control activity without disruption of normal activities. 3 Frequent pain. Discomfort relieved by oral medicines with occasional disruption of normal activities. 4 Frequent pain; frequent disruption of normal activities. Discomfort requires prescription narcotics for relief. 5 Severe pain. Pain not relieved by drugs and constantly disrupts normal activities. | Pain |
| 817 | Low back pain | Pain |
| 818 | Headaches | Pain |
| 819 | Sore muscles | Pain |
| 820 | Burning or sore eyes | Pain |
| 824 | Sore mouth/pain when swallowing | Pain |
| 828 | Abdominal ache | Pain |
| 927 | Pain intensity | Pain |
| 928 | Pain frequency | Pain |
| 929 | Pain distress | Pain |
| 1 | Do you have any trouble doing strenuous activities, like carrying a heavy shopping bag or a suitcase? | Physical |
| 2 | Do you have any trouble taking a long walk? | Physical |
| 3 | Do you have any trouble taking a short walk outside of the house? | Physical |
| 4 | Do you need to stay in bed or a chair during the day? | Physical |
| 110 | I am forced to spend time in bed | Physical |
| 188 | Because of your illness, how much physical strength have you lost? | Physical |
| 189 | Because of your illness, to what extent have you lost your endurance? | Physical |
| 190 | Because of your illness, to what extent do you feel unfit? | Physical |
| 251 | Vigorous activities, such as running, lifting heavy objects, participating in strenuous sports | Physical |
| 252 | Moderate activities, such as moving a table, pushing a vacuum cleaner, bowling, or playing golf | Physical |
| 253 | Lifting or carrying groceries | Physical |
| 254 | Climbing several flights of stairs | Physical |
| 255 | Climbing one flight of stairs | Physical |
| 256 | Bending, kneeling, or stooping | Physical |
| 257 | Walking more than a mile | Physical |
| 258 | Walking several blocks | Physical |
| 259 | Walking one block | Physical |
| 443 | How much are you active during the out-of work / resting hours? Please include sport, jogging, Socialevents. | Physical |
| 504 | MOBILITY - I have no problems in walking about; I have slight problems in walking about; I have moderate problems in walking about; I have severe problems in walking about; I am unable to walk about | Physical |
| 531 | How much physical strength do you have? | Physical |
| 532 | How is your overall physical well-being? | Physical |
| 560 | I can walk about only indoors. | Physical |
| 561 | I find it hard to bend. | Physical |
| 564 | I'm unable to walk at all. | Physical |
| 567 | I have trouble getting up and down stairs and steps. | Physical |
| 568 | I find it hard to reach for things. | Physical |
| 577 | I find it hard to stand for long (e.g., at the kitchen sink, waiting in a line). | Physical |
| 585 | I need help to walk about outside (e.g., a walking aid or someone to support me). | Physical |
| 603 | Walking up a flight of stairs | Physical |
| 604 | Running the length of a football field | Physical |
| 612 | Stay in your home, a nursing home, or hospital because of sickness, injury, or other health problem | Physical |
| 613 | Difficulty standing up for long periods | Physical |
| 614 | Difficulty lifting or carrying Body Images of approximately 10 pounds | Physical |
| 615 | Difficulty going up and down stairs | Physical |
| 616 | Difficulty walking | Physical |
| 617 | Difficulty stooping, bending or kneeling | Physical |
| 618 | Difficulty using hands and fingers | Physical |
| 619 | Difficult reaching with either or both arms | Physical |
| 628 | Using telephone - I = Able to look up numbers, dial telephone, and receive and make calls without help A = Able to answer telephone or dial operator in an emergency, but needs special telephone or help in getting numbers and/or dialing D = Unable to use telephone | Physical |
| 629 | Traveling - I = Able to drive own car or to travel alone on buses or in taxis A = Able to travel, but needs someone to travel with D = Unable to travel | Physical |
| 630 | Shopping - I = Able to take care of all food and clothes shopping with transportation provided A = Able to shop, but needs someone to shop with D = Unable to shop | Physical |
| 632 | Housework - I = Able to do heavy housework (i.e., scrub floors) A = Able to do light housework, but needs help with heavy tasks D = Unable to do any housework | Physical |
| 642 | To what extent (if at all) has your mobility (e.g., walking, driving a car, using public transport, etc.) been reduced by your problems or illness? | Physical |
| 682 | Describe how you feel right now: active | Physical |
| 730 | Describe how you feel right now: vigorous | Physical |
| 738 | Are you limited in any way in any activities because of any impairment or health problem? | Physical |
| 748 | Rate your overall physical health | Physical |
| 782 | SENSATION - 1 Able to see, hear, and speak normally for age. 2 Requires equipment to see or hear or speak. 3 Sees, hears, or speaks with limitations even with equipment. 4 Blind, deaf, or mute. | Physical |
| 783 | MOBILITY - 1 Able to walk, bend, lift, jump, and run normally for age. 2 Walks, bends, lifts, jumps, or runs with some limitations but does not require help. 3 Requires mechanical equipment (such as canes, crutches, braces, or wheelchair) to walk or get around independently. 4 Requires the help of another person to walk or get around and requires mechanical equipment as well. 5 Unable to control or use arms and legs. | Physical |
| 790 | ACTIVITY – During the past week the patient: 1) has been working or studying full-time, or nearly so, in usual occupation, or managing own household; or participating in unpaid or voluntary activities, whether retired or not. 2) has been working or studying in usual occupation or managing own household or participating in unpaid or voluntary activities; but requiring major assistance or a significant reduction in hours worked or a sheltered situation or was on sick leave. 3) has not been working or studying in any capacity and not managing own household. | Physical |
| 842 | Walk about house | Physical |
| 843 | Light housework/house jobs | Physical |
| 844 | Climb stairs | Physical |
| 845 | Heacy housework/house jobs | Physical |
| 846 | Walk out of doors | Physical |
| 890 | Do little | Physical |
| 891 | Take on a lot | Physical |
| 892 | Physically bad condition | Physical |
| 893 | Physically excellent condition | Physical |
| 894 | Very active | Physical |
| 895 | Do a lot in a day | Physical |
| 896 | Do very little in a day | Physical |
| 897 | Get little done | Physical |
| 676 | Describe how you feel right now: sorry for things done | Regret |
| 691 | Describe how you feel right now: spiteful | Regret |
| 706 | Describe how you feel right now: bitter | Regret |
| 729 | Describe how you feel right now: guilty | Regret |
| 868 | Guilt feelings | Regret |
| 6 | Were you limited in doing either your work or other daily activities? | Role |
| 7 | Were you limited in pursuing your hobbies or other leisure time activities? | Role |
| 124 | I am able to work (include work at home) | Role |
| 125 | My work (include work at home) is fulfilling | Role |
| 129 | I am enjoying the things I usually do for fun | Role |
| 191 | During the past 2 weeks, how often have you been able to complete your normal daily activities (school, work, household)? | Role |
| 192 | During the past 2 weeks, how often have you been able to take part in your usual patterns of leisure or recreational activities? | Role |
| 219 | I can enjoy a good book or radio or TV program | Role |
| 262 | Cut down the amount of time you spent on work or other activities. | Role |
| 263 | Accomplished less than you would like | Role |
| 264 | Were limited in the kind of work or other activities | Role |
| 265 | Had difficulty performing the work or other activities (for example, it took extra effort) | Role |
| 267 | Cut down the amount of time you spent on work or other activities | Role |
| 268 | Accomplished less than you would like | Role |
| 269 | Didn't do work or other activities as carefully as usual | Role |
| 298 | How often do you have to alter your lifestyle? Never = 0 (never), Rarely = <l/month, Sometimes = <l/week,_>l/month, Usually = <l/day, _>l/week, Always = _>l/day | Role |
| 359 | I feel that having cancer has interfered with my achievement of the most important goals I have set for myself. | Role |
| 391 | During the last two days It has been hard for me to get motivated to do my regular activities | Role |
| 392 | During the last two days I do very little in a day | Role |
| 400 | During the last two days I have had to restrict how much I try and do in a day | Role |
| 402 | During the last two days, I have had enough energy to read a newspaper/book or watch TV | Role |
| 429 | Did you adjust your activities to the availability of a toilet? | Role |
| 430 | Were you limited in your daily activities (e.g. work or house work) due to problems with your bowel movements? | Role |
| 442 | Are you active at home, including household activities? | Role |
| 444 | What about your working activities? | Role |
| 447 | Did you refuse excursion and/or travel because of your condition? | Role |
| 448 | Does it occur to you that you must interrupt your daily activity because of defecation problem or stoma-care? | Role |
| 506 | USUAL ACTIVITIES (e.g. work, study, housework, family or leisure activities) - I have no problems doing my usual activities; I have slight problems doing my usual activities; I have moderate problems doing my usual activities; I have severe problems doing my usual activities; I am unable to do my usual activities | Role |
| 523 | How useful do you feel? | Role |
| 589 | Is your persent state of health causing problems with your: Work? (that is, paid employment) | Role |
| 590 | Is your persent state of health causing problems with your: Looking after the home? (cleaning & cooking, repairs, odd jobs around the home etc) | Role |
| 594 | Is your persent state of health causing problems with your: Interests and hobbies? (sports, arts and crafts, do-it-yourself, etc.) | Role |
| 595 | Is your persent state of health causing problems with your: Vacations? (summer or winter vacations, weekends away, etc.) | Role |
| 631 | Preparing meals - I = Able to plan and cook full meals A = Able to prepare light foods, but unable to cook full meals alone D = Unable to prepare any meals | Role |
| 636 | To what extent (if at all) has your capacity to work or study effectively has been reduced by your problems or illness? | Role |
| 637 | To what extent (if at all) has your capacity to take part in enjoyable recreational activities been reduced by your problems or illness? | Role |
| 640 | To what extent (if at all) has your capacity to carry out routine chores (e.g., going shopping, maintaining the house, etc.) been reduced by your problems or illness? | Role |
| 734 | Does any impairment or health problem now keep you from working at a job or business? | Role |
| 735 | Are you limited in any kind or amount of work you can do because of any impairment or health problem? | Role |
| 736 | Does any impairment or health problem now keep you from doing any housework at all? | Role |
| 737 | Are you limited in any kind or amount of housework you can do because of any impairment or health problem? | Role |
| 740 | Because of any impairment or health problem, do you need the help of other persons in handling your routine needs, such as everyday household chores, doing necessary business, shopping or getting around for other purposes? | Role |
| 755 | How useful do you feel? | Role |
| 771 | To what degree has your illness and treatment interfered with your employment? | Role |
| 772 | To what degree has your illness and treatment interfered with your activities at home? | Role |
| 791 | DAILY LIVING – During the last week, the patient: 1) has been self-reliant in eating, washing, toileting and dressing; using public transport or driving own car. 2) has been requiring assistance (another person or special equipment) for daily activities and transport but performing light tasks. 3) has not been managing personal care nor light tasks and/or not leaving own home or institution at all. | Role |
| 847 | Go shopping | Role |
| 848 | Go to work | Role |
| 865 | Difficulty in returning to former roles | Role |
| 873 | Difficulty in pursuing the career of my choice | Role |
| 878 | Job discrimination | Role |
| 880 | Satisfaction with healthcare received | Satisfaction with care |
| 881 | Satisfaction with availability of doctors | Satisfaction with care |
| 882 | Satisfaction with nursing availability | Satisfaction with care |
| 883 | Satisfaction with continuity of care | Satisfaction with care |
| 884 | Satisfaction with personal qualities of nurses | Satisfaction with care |
| 885 | Satisfaction with communication with doctors and nurses | Satisfaction with care |
| 5 | Do you need help with eating, dressing, washing yourself or using the toilet? | Self-care |
| 260 | Bathing or dressing yourself | Self-care |
| 441 | Are you able to care for yourself? | Self-care |
| 505 | SELF-CARE - I have no problems washing or dressing myself; I have slight problems washing or dressing myself; I have moderate problems washing or dressing myself; I have severe problems washing or dressing myself; I am unable to wash or dress myself | Self-care |
| 575 | I find it hard to get dressed by myself. | Self-care |
| 596 | I like who I am | Self-care |
| 641 | To what extent (if at all) has your capacity to attend to your own daily needs (e.g., personal hygiene, personal appearance, dressing, etc.) been reduced by your problems or illness? | Self-care |
| 739 | Because of any impairment or health problem, do you need the help of other persons with your personal care needs, such as eating, bathing, dressing, or getting around the house? | Self-care |
| 786 | SELF-CARE - 1 Eats, bathes, dresses, and uses the toilet normally for age. 2 Eats, bathes, dresses, or uses the toilet independently with difficulty. 3 Requires mechanical equipment to eat, bathe, dress, or use the toilet independently. 4 Requires the help of another person to eat, bathe, dress, or use the toilet. | Self-care |
| 841 | Care for self | Self-care |
| 307 | How confident were you before your operation? | Self-esteem |
| 308 | How confident were you after your operation? | Self-esteem |
| 651 | I felt I was just as good as other people. | Self-esteem |
| 666 | I felt that people dislike me. | Self-esteem |
| 690 | Describe how you feel right now: unworthy | Self-esteem |
| 721 | Describe how you feel right now: effacious | Self-esteem |
| 725 | Describe how you feel right now: worthless | Self-esteem |
| 757 | Has your illness or treatment caused changes in your self concept (the way you see yourself)? | Self-esteem |
| 779 | To what extent has your illness made positive changes in your life? | Self-esteem |
| 803 | During the past 7 days about how much were you distressed or bothered by: Feeling of worthlessness | Self-esteem |
| 875 | Being treated as different from others | Self-esteem |
| 47 | To what extent were you interested in sex? | Sexual Function |
| 48 | To what extent were you sexually active (with or without intercourse)? | Sexual Function |
| 49 | Answer this question only if you have been sexually active: To what extent was sex enjoyable for you? | Sexual Function |
| 50 | Did you have difficulty getting or maintaining an erection? | Sexual Function |
| 51 | Did you have problems with ejaculation (e.g., so-called "dry ejaculation")? | Sexual Function |
| 52 | Did you have a dry vagina during intercourse? | Sexual Function |
| 53 | Did you have pain during intercourse? | Sexual Function |
| 100 | (Men) To what extent were you interested in sex? | Sexual Function |
| 101 | (Men) Did you have difficulty getting or maintaining an erection? | Sexual Function |
| 102 | (Women) To what extent were you interested in sex? | Sexual Function |
| 103 | (Women) Did you have pain or discomfort during intercourse? | Sexual Function |
| 117 | I am satisfied with my sex life | Sexual Function |
| 195 | To what extent has your sexual life been impaired (harmed) because of your illness? | Sexual Function |
| 228 | How often were you able to get an erection during sexual activity? | Sexual Function |
| 229 | When you had erections with sexual stimulation, how often were your erections hard enough for penetration? | Sexual Function |
| 230 | When you attempted intercourse, how often were you able to penetrate (enter) your partner? | Sexual Function |
| 231 | During sexual intercourse, how often were you able to maintain your erection after you had penetrated (entered) your partner? | Sexual Function |
| 232 | During sexual intercourse, how difficult was it to maintain your erection to completion of intercourse? | Sexual Function |
| 233 | How many times have you attempted sexual intercourse? | Sexual Function |
| 234 | When you attempted sexual intercourse, how often was it satisfactory for you? | Sexual Function |
| 235 | How much have you enjoyed sexual intercourse? | Sexual Function |
| 236 | When you had sexual stimulation or intercourse, how often did you ejaculate? | Sexual Function |
| 237 | When you had sexual stimulation or intercourse, how often did you have the feeling of orgasm or climax? | Sexual Function |
| 238 | How often have you felt sexual desire? | Sexual Function |
| 239 | How would you rate your level of sexual desire? | Sexual Function |
| 240 | How satisfied have you been with your overall sex life? | Sexual Function |
| 241 | How satisfied have you been with your sexual relationship with your partner? | Sexual Function |
| 242 | How do you rate your confidence that you could get and keep an erection? | Sexual Function |
| 243 | How do you rate your confidence that you could get and keep an erection? | Sexual Function |
| 244 | When you had erections with sexual stimulation, how often were your erections hard enough for penetration? | Sexual Function |
| 245 | During sexual intercourse, how often were you able to maintain your erection after you had penetrated (entered) your partner? | Sexual Function |
| 246 | During sexual intercourse, how difficult was it to maintain your erection to completion of intercourse? | Sexual Function |
| 247 | When you attempted sexual intercourse, how often was it satisfactory for you? | Sexual Function |
| 381 | If you were to spend the rest of your life with your sexual condition the way it is now, how would you feel about that? | Sexual Function |
| 432 | Were you limited in your sexual activities (with or without sexual intercourse) due to problems with your bowel movements? | Sexual Function |
| 471 | Did your sexual function change on a negative manner since the surgical intervention? | Sexual Function |
| 476 | (Men) Do you have any erectile dysfunction? | Sexual Function |
| 477 | (Men) Is there any problem with ejaculation compared to the pre-operative situation? | Sexual Function |
| 478 | (Women) Do you have any pain during cohabitation? Do you experience the lack of desire, arousal and /or orgasm since? | Sexual Function |
| 593 | Is your persent state of health causing problems with your: Sex life? | Sexual Function |
| 770 | Is your sexuality impacted by your illness? | Sexual Function |
| 822 | Decreased sexual interest | Sexual Function |
| 854 | Being less physically able to have sexual intercourse | Sexual Function |
| 879 | Concern about being physically unable to have children | Sexual Function |
| 8 | Were you short of breath? | SoB |
| 343 | During the past 7 days, how much were you distressed by: Trouble getting your breath | SoB |
| 472 | Do you suffer of some chest pain, dyspnoea or other respiratory disturbance? | SoB |
| 627 | Shortness of breath, trouble breathing even when not exercising or working hard | SoB |
| 826 | Shortness of breath | SoB |
| 26 | Has your physical condition or medical treatment interfered with your family life? | Social |
| 27 | Has your physical condition or medical treatment interfered with your Socialactivities? | Social |
| 99 | (No stoma) Did you feel embarrassed because of your bowel movement? | Social |
| 106 | Because of my physical condition, I have trouble meeting the needs of my family | Social |
| 111 | I feel close to my friends | Social |
| 112 | I get emotional support from my family | Social |
| 113 | I get support from my friends | Social |
| 114 | My family has accepted my illness | Social |
| 115 | I am satisfied with family communication about my illness | Social |
| 116 | I feel close to my partner (or the person who is my main support) | Social |
| 194 | To what extent have your personal relations with people close to you (family or friends) worsened because of your illness? | Social |
| 270 | During the past 4 weeks, to what extent has your physical health or emotional problems interfered with your normal Socialactivities with family, friends, neighbors, or groups? | Social |
| 283 | During the past 4 weeks, how much of the time has your physical health or emotional problems interfered with your Socialactivities (like visiting with friends, relatives, etc.)? | Social |
| 357 | I feel that my relationships with other people have not been negatively affected by my cancer experience. | Social |
| 431 | Were you limited in your Socialactivities (e.g. family visits, visits to the theatre, or eating out) due to problems with your bowel movements? | Social |
| 445 | What is the relation of your family members and friends to you? | Social |
| 446 | Did you abandon some Socialactivities as good company, meeting with friends, going out, cinema, theatre etc.? | Social |
| 521 | How distressing has your illness been for your family? | Social |
| 526 | Is support from friends/family sufficient? | Social |
| 550 | Do you have difficulty meeting new people? | Social |
| 559 | I feel lonely. | Social |
| 565 | I'm finding it hard to make contact with people. | Social |
| 571 | I feel there is nobody that I am close to. | Social |
| 580 | I feel I am a burden to people. | Social |
| 584 | I'm finding it hard to get along with people. | Social |
| 591 | Is your persent state of health causing problems with your: Sociallife? (going out, seeing friends, going to the movies, etc.) | Social |
| 592 | Is your persent state of health causing problems with your: Home life? (that is, relationships with other people in your home) | Social |
| 597 | I am not an easy person to get along with | Social |
| 601 | I am happy with my family relationships | Social |
| 602 | I am comfortable being around people | Social |
| 610 | Socialize with other people (talk or visit with friends or relatives) | Social |
| 611 | Take part in social, religious, or recreation activities (meetings, church, movies, sports, parties) | Social |
| 635 | To what extent (if at all) has your capacity to be involved in satisfying relationships (with family, friends, workmates) been reduced by your problems or illness? | Social |
| 638 | To what extent (if at all) has your capacity to adequately fulfil Socialor religious obligations been reduced by your problems or illness? | Social |
| 639 | To what extent (if at all) has your capacity to meet family obligations or expectations been reduced by your problems or illness? | Social |
| 660 | I talked less than usual. | Social |
| 661 | I felt lonely. | Social |
| 662 | People were unfriendly. | Social |
| 668 | Describe how you feel right now: friendly | Social |
| 702 | Describe how you feel right now: lonely | Social |
| 767 | How distressing has illness been for your family? | Social |
| 768 | Is the amount of support you receive from others sufficient to meet your needs? | Social |
| 769 | Is your continuing health care interfering with your personal relationships? | Social |
| 773 | How much isolation do you feel is caused by your illness or treatment? | Social |
| 793 | SUPPORT – During the last week: 1) the patient has been having good relationships with others and receiving strong support from at least one family member. 2) support received or perceived has been limited from family and friends and/or by the patient’s condition. 3) support from family and friends occurred infrequently or only when absolutely necessary or the patient was unconscious. | Social |
| 802 | During the past 7 days about how much were you distressed or bothered by: Feeling afraid to go out of your home alone | Social |
| 804 | During the past 7 days about how much were you distressed or bothered by: Feeling lonely even when you are with people | Social |
| 807 | During the past 7 days about how much were you distressed or bothered by: Feeling lonely | Social |
| 870 | Problems communicating with my spouse or partner | Social |
| 876 | Problems with family/children | Social |
| 540 | Support from personal spiritual activities sufficient? | Spiritual |
| 541 | Support from religious activities sufficient? | Spiritual |
| 544 | Do you sense a reason for being alive? | Spiritual |
| 775 | How important to you is your participation in religious activities such as praying, going to church? | Spiritual |
| 776 | How important to you are other spiritual activities such as meditation? | Spiritual |
| 777 | How much has your spiritual life changed as a result of cancer diagnosis? | Spiritual |
| 780 | Do you sense a purpose/mission for your life or a reason for being alive? | Spiritual |
| 62 | Were you afraid that other people would be able to hear your stoma? | Stoma Problems |
| 63 | Were you afraid that other people would be able to smell your stools? | Stoma Problems |
| 64 | Were you worried about possible leakage from the stoma bag? | Stoma Problems |
| 65 | Did you have problems with caring for your stoma? | Stoma Problems |
| 66 | Was your skin around the stoma irritated? | Stoma Problems |
| 67 | Did you feel embarrassed because of your stoma? | Stoma Problems |
| 68 | Did you feel less complete because of your stoma? | Stoma Problems |
| 87 | (Stoma) Have you had unintentional release of gas/flatulence from your stoma bag? | Stoma Problems |
| 88 | (Stoma) Have you had leakage of stools from your stoma bag? | Stoma Problems |
| 89 | (Stoma) Have you had sore skin around your stoma? | Stoma Problems |
| 90 | (Stoma) Did frequent bag changes occur during the day? | Stoma Problems |
| 91 | (Stoma) Did frequent bag changes occur during the night? | Stoma Problems |
| 92 | (Stoma) Did you feel embarrassed because of your stoma? | Stoma Problems |
| 93 | (Stoma) Did you have problems caring for your stoma? | Stoma Problems |
| 139 | (Stoma) I am embarrassed by my ostomy appliance | Stoma Problems |
| 140 | (Stoma) Caring for my ostomy appliance is difficult | Stoma Problems |
| 482 | (Stoma) Do you have a problem with the control of intestinal gases? | Stoma Problems |
| 483 | (Stoma) Do you experience any faecal incontinence? | Stoma Problems |
| 484 | (Stoma) How often do you experience faecal incontinence? | Stoma Problems |
| 485 | (Stoma) Are you able to control your stool for at least ten minutes following the first sensation? | Stoma Problems |
| 486 | (Stoma) Do you have any problem with defecation? Does it occur to you, that there is a need for repeated defecation shortly after the usual one? | Stoma Problems |
| 487 | (Stoma) Do you wear any incontinence care product? | Stoma Problems |
| 488 | (Stoma) Do you have defecation without previous sensation? | Stoma Problems |
| 489 | (Stoma) Do you have pain during defecation? | Stoma Problems |
| 490 | (Stoma) Do you have any peri-anal skin disorder as irritation, itching or inflammation? | Stoma Problems |
| 491 | How do you feel with your stoma? | Stoma Problems |
| 492 | Besides your family who else are aware of your stoma? | Stoma Problems |
| 493 | In your opinion how well you are educated in stoma care? | Stoma Problems |
| 494 | Number of bag changes per day? | Stoma Problems |
| 495 | Number of base plates used during the week? | Stoma Problems |
| 496 | Do you experience some smelling around the bag – except the period of the usual manipulation when you change it? | Stoma Problems |
| 497 | Are there any sounds around your stoma? | Stoma Problems |
| 498 | Do the sound and odour events disturb you? | Stoma Problems |
| 499 | Did you experience some leakage around the bag? | Stoma Problems |
| 500 | Does any skin disorder as swelling, inflammation, coloration, secretion, bleeding - disturb you? | Stoma Problems |
| 501 | Did a hernia develop in the stoma site? | Stoma Problems |
| 502 | Are you a club member in an ILCO club or other club for ostomates or handicapped people? | Stoma Problems |
| 510 | Has ostomy interfered with Socialactivities? | Stoma Problems |
| 511 | How much isolation is caused by your ostomy? | Stoma Problems |
| 512 | Has ostomy interfered with personal relationships? | Stoma Problems |
| 513 | How much does ostomy interfere with travel? | Stoma Problems |
| 514 | Difficult to adjust to ostomy? | Stoma Problems |
| 515 | Has ostomy interfered with rec-sports activities? | Stoma Problems |
| 516 | Has ostomy interfered with ability to be intimate? | Stoma Problems |
| 517 | How difficult is it to care for ostomy? | Stoma Problems |
| 518 | How much are you embarrassed by ostomy? | Stoma Problems |
| 519 | How difficult is it to look at your ostomy? | Stoma Problems |
| 527 | Do you have enough privacy at home for ostomy care? | Stoma Problems |
| 529 | Do you have enough privacy when traveling for ostomy care? | Stoma Problems |
| 537 | Problem with skin surrounding ostomy? | Stoma Problems |
| 538 | Problem with leaking from pouch or around appliance? | Stoma Problems |
| 545 | Has having an ostomy made positive changes in your life? | Stoma Problems |
| 42 | Did food and drink taste different from usual? | Taste |
| 80 | Have you had problems with your sense of taste? | Taste |
| 481 | Did you suffer of some gum problem in connection with this treatment? | Taste |
| 323 | Do you have a feeling of residual stool after evacuation? Always, Occasionally, Never | Tenesmus |
| 360 | Fractionated defecation | Tenesmus |
| 366 | Incomplete evacuation | Tenesmus |
| 375 | How severe have each of these symptoms been in the last 2 weeks? Incomplete bowel movement, like you didn't "finish" | Tenesmus |
| 377 | How severe have each of these symptoms been in the last 2 weeks? Bowel movements that were too small | Tenesmus |
| 427 | Did you feel that your bowels were not empty after your bowel movement? | Tenesmus |
| 428 | After you had a bowel movement, did you have to return to the toilet within 1 h for a bowel movement? | Tenesmus |
| 193 | During the past 2 weeks, how much have you been troubled by the medical treatment of your illness? | Treatment problems |
| 322 | Do you need laxatives? Always, Occasionally, Never | Treatment problems |
| 325 | Do you need enemas, suppositories or a catheter for evacuation? Always, Occasionally, Never | Treatment problems |
| 362 | Pads needed | Treatment problems |
| 363 | Enemas needed | Treatment problems |
| 364 | Antidiarrheic drugs needed | Treatment problems |
| 438 | Have you used medicines to thicken your stools? | Treatment problems |
| 466 | To regulate the stool do you take some medicine? | Treatment problems |
| 467 | Do you get enemas? | Treatment problems |
| 555 | I take pills to help me sleep. | Treatment problems |
| 633 | Taking medicine - I = Able to prepare and take medications in the right dose at the right time A = Able to take medications, but needs reminding or someone to prepare them D = Unable to take medications | Treatment problems |
| 719 | Describe how you feel right now: deceived | Trust |
| 722 | Describe how you feel right now: trusting | Trust |
| 31 | Did you urinate frequently during the day? | Urinary Frequency |
| 32 | Did you urinate frequently during the night? | Urinary Frequency |
| 69 | Did you urinate frequently during the day? | Urinary Frequency |
| 70 | Did you urinate frequently during the night? | Urinary Frequency |
| 470 | Did your bladder function change since the surgical intervention? | Urinary Frequency |
| 71 | Have you had any unintentional release (leakage) of urine? | Urinary Incontinence |
